# Photoactive PFA Coating through Fluorophilic Interactions for Continuous Flow Photochemistry

**DOI:** 10.1021/jacsau.5c00804

**Published:** 2025-11-25

**Authors:** Jesús Castro-Esteban, John H. Dunlap, Benedikt S. Schreib, Peter Mirau, Christopher A. Crouse, Timothy M. Swager, Luke A. Baldwin

**Affiliations:** † Department of Chemistry, 2167 Massachusetts Institute of Technology, Cambridge, Massachusetts 02139, United States; ∥ Materials and Manufacturing Directorate, Air Force Research Laboratory, Wright-Patterson AFB, Dayton, Ohio 45433, United States; § AV, Inc, Dayton, Ohio 45432, United States

**Keywords:** photochemistry, perfluoroalkoxy (PFA), photocatalysts, functionalized coil flow reactors, fluorophilic interactions

## Abstract

The design of flow reactors for heterogeneous photocatalysis is key to enhancing the control, efficiency, and scalability of chemical reactions. However, conventional designs such as slurry reactors and fixed bed reactors often suffer from poor light penetration, challenging catalyst attachment to the support, and difficult separations. We report an efficient and robust methodology for the functionalization of perfluoroalkoxy (PFA) coil reactors with different fluorinated photocatalysts [a perylene diimide (**F-PDI**) and poly­(*p*-phenylene ethynylene) polymers (**PPEST** and **POLPDI**)] through fluorophilic interactions. We have evaluated the efficiency of photocatalyst-functionalized coil reactors in continuous flow experiments through the [2 + 2] photocycloaddition of 9-vinylcarbazole (**VCZ**) using blue and green light (440 and 525 nm). The conversion of **VCZ** to the product 1,2-trans-dicarbazylcyclobutane (***t*** **-DCZCB**) was continuously monitored by in-line nuclear magnetic resonance (NMR) spectroscopy, and we found that **PPEST** was the most robust photocatalyst coating of those studied, leading to high conversions with different lamp powers and residence times. Further experiments proved that **PPEST**-functionalized coil reactors were stable and efficient after 18 h of continuous flow with conversions from around 50 to 75%.

## Graphical abstract

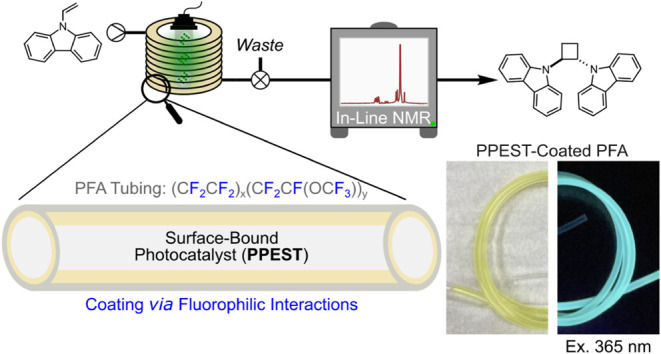

## Introduction

The development of continuous flow photochemistry has significantly improved the efficiency, safety, and sustainability of chemical processes in both academia and industry.^1–3^ Flow photochemistry overcomes traditional limitations of batch systems such as precise control over reaction parameters, uniform light exposure, shorter reaction times, scalability, lower energy consumption, reduced waste generation, and the ability to incorporate automated analysis.^4–6^ Photochemical reactions in flow involving catalytic elements may be categorized by how the photocatalyst (PC) is employed.^7–9^ Homogeneous flow photocatalysis features reagents and catalysts in the same phase but often necessitates an additional purification stage for PC separation, and catalysts are generally not recovered for reuse. Conversely, in heterogeneous flow photocatalysis, the reagents and catalysts are in different phases (e.g., liquid–solid), often with the PC supported within a photoreactor through which reagents flow.^7^ Heterogeneous photocatalysis in flow offers a more scalable and sustainable method for conducting photochemical reactions by avoiding extra separation steps and enabling catalyst recycling.

The configuration of flow reactors significantly impacts the control, efficiency, and scalability of chemical reactions in heterogeneous photocatalysis. These photoreactors are categorized by their catalytic form, including fixed bed, slurry, and coated designs.^10,11^ In fixed bed reactors, the photocatalyst is supported on conventional materials like silica, zeolite, glass beads, or polymers, and then packed into columns (Figure 1a).^12^ In slurry reactors, the PC (in small particle form) is suspended in the reaction mixture which usually involves gas and liquid mixtures (Figure 1b).^13^ In coated reactors, however, the catalyst is immobilized on the surface of microfluidic channels (Figure 1c).^11,14^ To be successful, coated photoreactors typically necessitate surface modification and functionalization to achieve proper loading of the catalyst and good adherence to the reactor material. This process can often involve intricate designs for efficient operation and can be challenging to implement in specific situations. Recently, Lanterna and coworkers reported the functionalization of glass beads with perylene diimides (PDI) and explored their role as heterogeneous photocatalysts in packed bed coils.^15^ Coil reactors are a cost-effective reactor design in flow chemistry, which can be utilized for both homogeneous and heterogeneous catalysis, although their common application is for single-phase reactions in solution.^16^ In 2005, Pohl and coworkers introduced fluorous interations using perfluoroalkyl chains to noncovalently anchor carbohydrates onto fluorinated surfaces (PTFE/epoxy mixtures).^17^ Coil reactors are often made from fluoropolymers, such as perfluoroalkoxy (PFA), due to their high chemical tolerance, temperature stability, resistance to fouling, ultraviolet (UV) light stability, and pressure resistance. Although these features can make flouropolymers challenging to functionalize, we previously reported the coating of PFA tubing with a photoactive fluorinated polymer that was functionalized after polymerization via C–H fluoroalkylation.^18^ While this promising coating methodology harnessed the fluorine–fluorine interactions of the PFA tubing with perfluoroalkyl chains to create immobilized catalysts, the coating was somewhat irregular, and flow experiments resulted in leaching of the photocatalyst.

**Figure 1:**
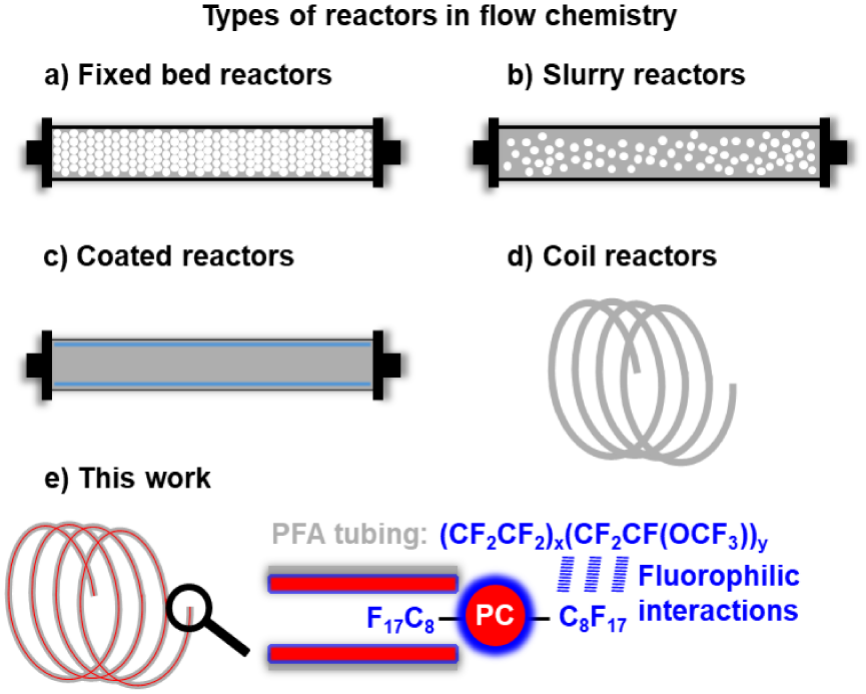
Types of reactors in flow chemistry: (a) Fixed bed reactor. (b) Slurry reactor. (c) Coated reactor. (d) Coil reactor. (e) Dye-functionalized PFA coil reactor.

Herein, we report an efficient and robust methodology for the functionalization of PFA tubing with additional photocatalysts. The fluorous version of a perylene diimide (**F-PDI**) and fluorinated poly­(*p*-phenylene ethynylene) (PPE) polymers containing photoactive groups (**PPEST** and **POLPDI**) were synthesized. Each photocatalyst features perfluoroalkyl chains on the periphery to increase their fluorine content and enhance the fluorophilic interactions with PFA, while decreasing their solubility in common organic solvents to reduce leaching. Furthermore, PFA coil reactors were functionalized and evaluated by exploring their efficiency in the [2 + 2] photocycloaddition of 9-vinylcarbazole (**VCZ**) as a function of LED lamp wavelength, radiant power, and residence time. To investigate the stability of the photocatalyst coating over extended periods of continuous flow, benchtop 60 MHz ^1^H NMR spectroscopy, coupled with an in-line flow cell, was utilized to continuously monitor the reaction over 18 h by directly analyzing the reaction mixture as it flowed through the instrument, providing a clear and continuous assessment of the reaction state. Data acquisition was automated and controlled via Python, while an open-source Python module (nmrglue) was used to automate data processing, which allowed for dynamic tracking of reactant consumption and product formation under steady-state conditions.

## Results and Discussion

### Design of Photocatalysts

Photocatalyst design is a key factor for PFA tubing functionalization. To have efficient fluorine–fluorine interactions between the photocatalysts and PFA tubing, we designed photoactive compounds with high fluorine content (>30 wt % F). Incorporating perfluoroalkyl chains on the periphery of the photocatalysts limited their solubility in common organic solvents and reduced leaching during flow experiments. To demonstrate that higher F content in the PC dyes would enhance PFA functionalization efficiency, the coating efficiency of small molecules and polymers was compared. Figure 2 shows the three photocatalysts used in this work: a fluorinated perylene diimide (**F-PDI**) and two polymers containing photoactive groups such as perfluoroalkyl terephthalate (**PPEST**) and PDI (**POLPDI**).

**Figure 2:**
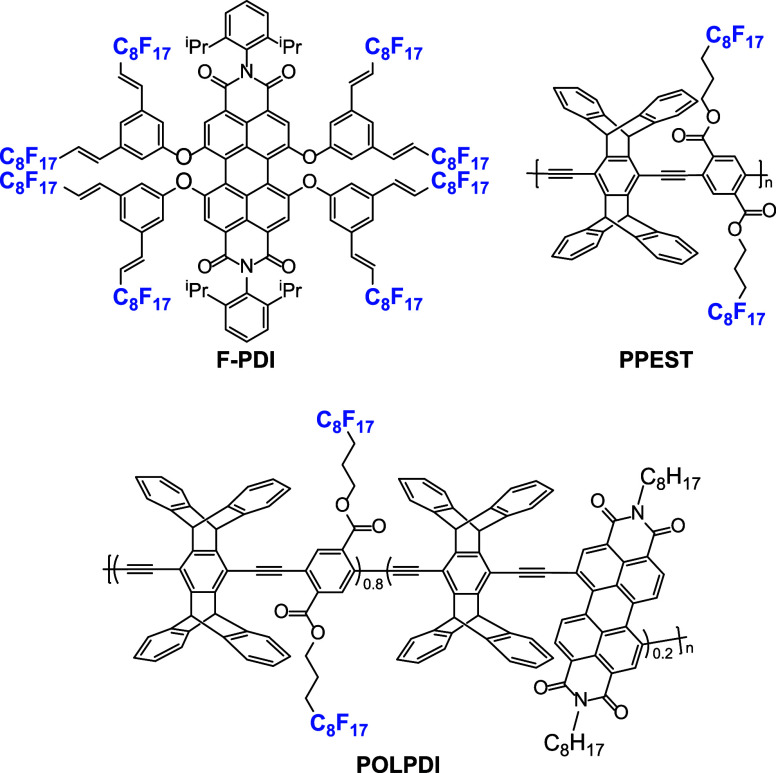
Photocatalysts used in this work.

Perylene diimides are effective photocatalysts due to their strong light absorption and stable redox states, acting as both electron donors and acceptors in photocatalytic reactions.^19–22^ For these reasons, **F-PDI**, which was previously reported by our group,^23^ was used. This dye, synthesized through fluorous Heck coupling, has a high fluorine content (56 wt % F) due to eight vinyl perfluoroalkyl chains. However, despite its high fluorine content, it has some solubility in organic solvents of interest for photochemical reactions (e.g., acetone, toluene, and chloroform). To mitigate the loss of the catalyst under continuous flow conditions in solution, it is advantageous to use photocatalysts that are insoluble in the reaction solvent. To obtain photocatalysts with lower solubility, we synthesized fluorinated poly­(*p*-phenylene ethynylene) (PPE) polymers containing pentiptycene and photoactive monomers.^24,25^ The PPE polymers were synthesized by Sonogashira polymerization between dialkyne pentiptycene and perfluoroalkyl dibromo terephthalate (**PPEST**) as well as polymerization between dialkyne pentiptycene, perfluoroalkyl dibromo terephthalate, and dibromo perylene diimide (**POLPDI**). For more synthetic details, see the Supporting Information. Electron-deficient ester monomers were strategically selected to oxidatively generate radicals under photoredox conditions. **PPEST** and **POLPDI** have high fluorine content (41.4 and 35.2 wt % F, respectively) with limited solubility in common organic solvents (i.e., insoluble in MeOH, acetone, acetonitrile, and moderate solubility in CHCl_3_, CH_2_Cl_2_, and THF). **POLPDI** with a monomer ratio of 0.8:0:2 (perfluoroalkyl dibromo terephthalate:PDI) was selected based on our previous work,^18^ in which 20% photoactive monomer was sufficient to efficiently perform photocatalytic transformations as well as to keep the weight % fluorine as close as possible to that of **PPEST**. The relative molecular weight (Mn) and polydispersity (Đ) of the polymers were determined by gel permeation chromatography (GPC) in THF (Figure S2). Due to the moderate solubility of the polymers in THF, only the soluble part was analyzed and exhibited Mn = 26.5 kDa for **PPEST** and Mn = 12.8 kDa for **POLPDI**.

### PFA Tubing Functionalization

Coil reactors made from perfluoroalkoxy (PFA) tubing are ubiquitous in flow chemistry, represented by the chemical formula (CF_2_CF_2_)_*x*_(CF_2_CF­(OCF_3_))_*y*_. The perfluoroalkyl side chains on the photocatalysts produce fluorophilic interactions with PFA tubing. To functionalize PFA tubing, the material was heated above its glass transition temperature (*T*_g_)^26^ to increase interdiffusion of the fluorous side chains and promote uniform coatings with the fluorinated dyes. Hence, the PFA tubing was heated at 120 °C in an oil bath and filled with a hot solution of the photocatalyst in anisole (5 mg·mL^–1^ for **F-PDI** and 2.5 mg·mL^–1^ for the polymers). After 10 min of heating at 120 °C, the tubing (filled with the PC solution) was allowed to cool over the course of 1 h. Finally, the solution was drained, the tubing was rinsed with acetonitrile (MeCN), and dried in a vacuum desiccator for 16 h.

In order to effectively coat PFA, the dyes require good solubility in the solvent to achieve a homogeneous coating. Figure 3 shows the results after coating the PFA tubing with three different PCs. As the images highlight, PFA functionalization with **F-PDI** gave a somewhat irregular red coating with red emission due to the low solubility of the dye in anisole at room temperature and 120 °C (Figure 3a). Alternative organic solvents such as *o*-dichlorobenzene and *m*-xylenes were also tested, but the solubility of **F-PDI** was still very poor, and similar coatings were observed. The PFA functionalization with **PPEST**, however, gave a very homogeneous yellow coating with blue-green emission despite the polymer being only moderately soluble in anisole or *o*-dichlorobenzene (Figure 3b). **POLPDI** demonstrated greater solubility in anisole, producing a very homogeneous dark yellow coating with a red emission (Figure 3c). The necessity of perfluoroalkyl chains for this coating approach was confirmed by testing a nonfluorinated perylene diimide derivative on PFA tubing (Figure S9), which showed no dye adhesion, highlighting the crucial role of fluorous chains for efficient PFA tubing functionalization without surface pretreatment. In addition, the extension of this coating methodology to other fluoropolymers was successfully demonstrated with the coating of **PPEST** onto PTFE tubing (Figure S10).

**Figure 3:**
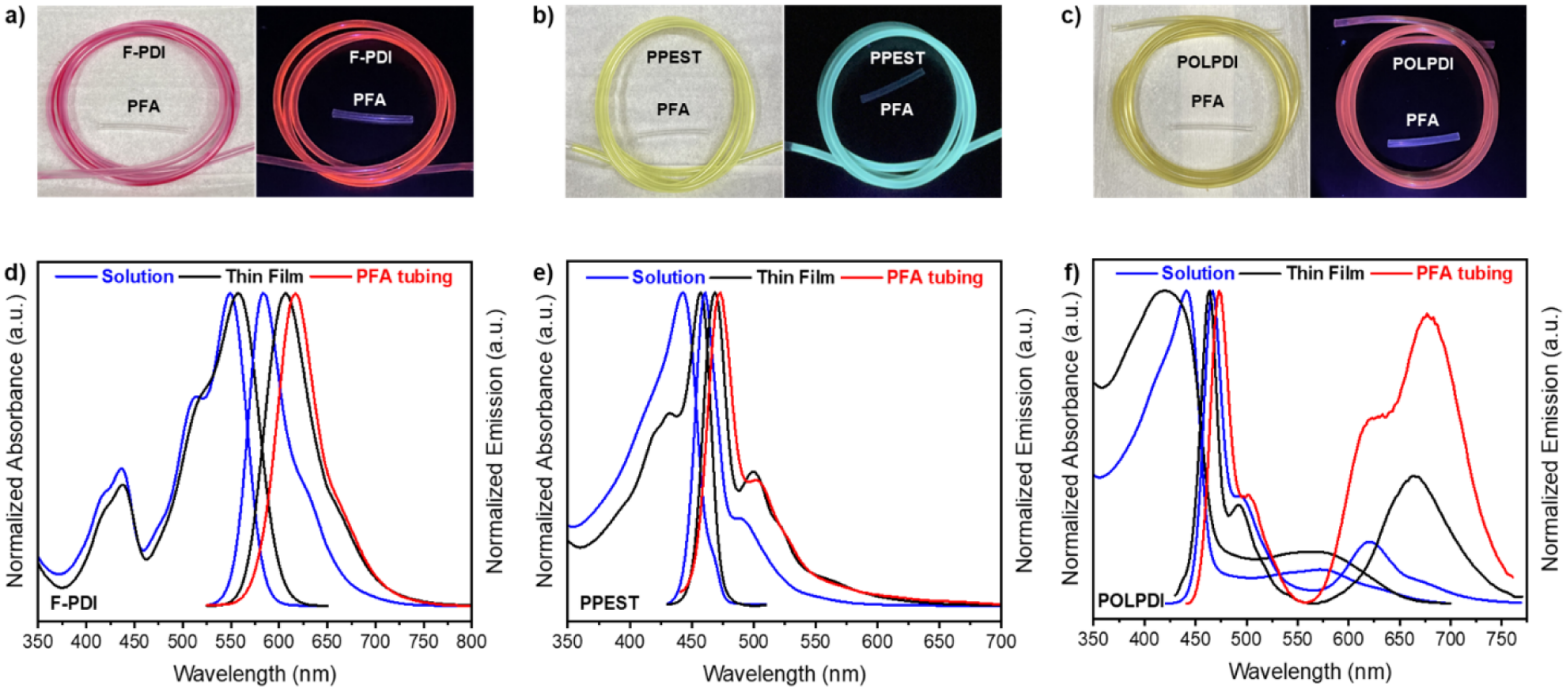
(a)–(c) Coating of PFA tubing with **F-PDI**, **PPEST**, and **POLPDI**, respectively. Left, view of the tubing with natural light. Right, view of the emission under irradiation with 365 nm light. (d)–(f) UV–vis and fluorescence spectra of **F-PDI**, **PPEST,** and **POLPDI**, respectively.

### Optical Properties

The ultraviolet–visible (UV–vis) absorption and fluorescence spectra for **F-PDI**, **PPEST**, and **POLPDI** were collected in benzotrifluoride (PhCF_3_) solutions and spin-cast films. In addition, the emission of functionalized PFA tubing was also measured using a bifurcated fiber optic accessory. Table 1 presents the corresponding optical data. The UV–vis spectra of **F-PDI** and **PPEST** both exhibit a broad absorption band in the visible range, making them strong candidates for photocatalytic applications. In contrast, **PPEST** shows a narrower absorption, which limits the application of this dye to some degree. However, due to the absorption and emission spectra overlap, the material can still be utilized with green light irradiation (LEDs centered at 525 nm). The emission of **F-PDI** in solution was blue-shifted and narrower as a thin film and on PFA tubing, which was expected due to intermolecular interactions in the solid state. For **PPEST**, the solid-state emission was slightly red-shifted relative to that of PhCF_3_ solutions; however, the thin film and PFA tubing emissions overlapped in this case. **POLPDI** exhibited characteristics similar to those of both **PDI** and **PPEST**, displaying both emission bands. Notably, there is a further increase of the red emission band in solid-state spectra from thin films to PFA tubing, reflecting increased conformational order and conjugation lengths produced by intermolecular organization.

**Table 1:** Optical Properties for F-PDI, PPEST, and POLPDI

	Absorption (λ_max_)		Emission (λ_max_)		
	PhCF_3_	Thin Film	PhCF_3_	Thin Film	PFA
F-PDI	549 nm	557 nm	583 nm	607 nm	617 nm
PPEST	443 nm	457 nm	461 nm	469 nm	473 nm
POLPDI	441 nm	419 nm	467 nm	464 nm	473 nm

### Photocatalyst Quantification in the Tubing

The molar absorptivity of each dye was determined by the Beer–Lambert law in benzotrifluoride solutions. Similar values for **F-PDI** and **POLPDI** (ε_549_ = 60,019 M^–1^ cm^–1^ and ε_441_ = 57,789 M^–1^ cm^–1^, respectively) were observed and higher for **PPEST** (ε_443_ = 83,700 M^–1^ cm^–1^). With this data, the amount of photocatalyst per centimeter of tubing was quantified by coating a section of standard PFA tubing (see Supporting Information). While the inner and outer diameters of continuous flow tubing may differ slightly depending on researcher needs, to quantify the efficiency of photocatalyst coating, 5 sections of PFA tubing (ID: 1.6 mm, OD: 3.175 mm, from 1, 2, and 4 mL coils) with known length (between 3 and 5 cm) were selected. Then, the tubing was immersed in benzotrifluoride to dissolve all the PC, and the UV–vis spectrum for each test was measured to quantify the amount of dye released into solution. From these tests, it was apparent that **F-PDI** yielded a significantly higher amount of dye per cm of tubing (26.37 ± 12.79 μg·cm^–1^) compared to **PPEST** and **POLPDI** (0.095 ± 0.039 and 0.354 ± 0.110 μg·cm^–1^, respectively). We further determined that the polymer coatings require only a few micrograms of conjugated polymer per centimeter to produce a uniform photocatalyst distribution and highly emissive tubing. Additional dye leaching studies for the three dyes in common organic solvents are included in the Supporting Information.

### Continuous Flow Experiments

Following our methodology to prepare dye-functionalized PFA tubing for synthetic reactions, 2 mL coiled tube reactors (1.3 mm I.D., 1.6 mm O.D.) were coated with **F-PDI**, **PPEST**, and **POLPDI** (Figure S6). The photocatalytic efficiency of the catalysts was then tested using a [2 + 2] cycloaddition reaction of 9-vinylcarbazole (**VCZ**) as a model reaction. This is an important transformation to access cyclobutanes, which feature prominently in natural products and are of increasing interest to the pharmaceutical industry. This photocatalytic dimerization has been studied in both batch and continuous flow, making it an ideal reaction to understand the efficiency of immobilized fluorous photocatalysts in flow.^27^ Other photocatalytic transformations were also studied, and the results are discussed in the Supporting Information.

The [2 + 2] photocycloaddition of **VCZ** was performed in continuous flow using a Vaportec R-Series modular flow chemistry system equipped with a UV-150 photochemical reactor, represented schematically in Figure 4. In brief, the coated PFA tubing was wrapped around a scaffold and placed inside the reactor module, where it was positioned between the LED lamp and a dichroic mirror. A solution of **VCZ** (10 mg·mL^–1^) in a 1:5 v/v acetone/MeCN mixture was flowed through the reactor at a given flow rate, and the reaction was initiated by light from a monochromatic LED lamp (440 or 525 nm). To ensure steady-state conditions were reached, three reactor volumes of the reaction mixture were passed to waste before collecting aliquots of the product mixture using a fraction collector. The product was concentrated, and ^1^H NMR spectroscopy measurements were taken to quantify the % conversion of **VCZ** to the product 1,2-trans-dicarbazylcyclobutane (***t*** **-DCZCB**). To study the photocatalytic dimerization, the effects of polymer catalyst, lamp power, and residence time (τ_res_) on the conversion of **VCZ** to ***t*** **-DCZCB** were investigated. Full experimental details are provided in the Supporting Information.

**Figure 4:**
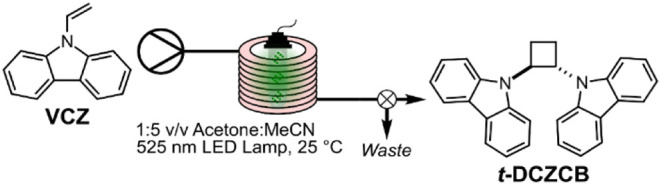
Schematic of continuous flow synthesis of ***t*-DCZCB** by **VCZ** photocycloaddition with **F-PDI** functionalized PFA tubing.

Prior to investigating the parameters mentioned above, the conversion of **VCZ** was tested as a function of lamp emission wavelength in the absence of a photocatalyst. Irradiation by the blue LED (440 nm) led to 30.1% conversion to ***t*** **-DCZCB** as well as byproduct formation, as seen by ^1^H NMR spectra in Figure S23. However, negligible conversion was observed by using the green LED (525 nm). As a result of the significant **VCZ** conversion under blue light, reactions utilizing 525 nm irradiation were selected to ensure conversion occurs through photocatalyst action.

To determine the effects of radiant lamp power on photoconversion, three relative power settings were selected: 10%, 46%, and 99% (maximum power), which correspond to 0.3, 1.38, and 3.0 W, respectively (Figure 5a). We hypothesized that increases in power output would boost ***t*** **-DCZCB** formation as more photons would be available to interact with the photocatalyst. Comparing the photoconversion at the lowest radiant power to the higher powers for **F-PDI** and **PPEST**, an increase in conversion was indeed observed (Figure 5a), with **VCZ** conversion nearly doubling for **F-PDI** and improving 5× for **PPEST**. Interestingly, the amount conversion at 1.38 and 3.0 W for these two catalysts is nominally the same for a given catalyst but also above 90%. In the case of **POLPDI**, the reaction was only tested at maximum power. After a single reaction (15.15 min τ_res_), the **POLPDI** polymer coating was no longer intact, with significant loss of catalyst throughout the tubing where direct irradiation occurred. This observation could be due to either poor polymer-tubing interactions, or photobleaching. Visual analysis after repeated irradiation and photocatalysis showed that the **F-PDI** coating on the tubing decreased in areas of direct irradiation, but the coating demonstrated greater resistance to degradation than **POLPDI**. Conversely, the photocatalytic coating composed of **PPEST** exhibited a markedly superior level of resistance and durability under the operational conditions. Visual examination of the **PPEST** coating after the experimental use period indicated that it remained intact, displaying no significant signs of degradation or damage. This observation underscores the inherent robustness and resilience of the **PPEST** photocatalyst coating.

**Figure 5:**
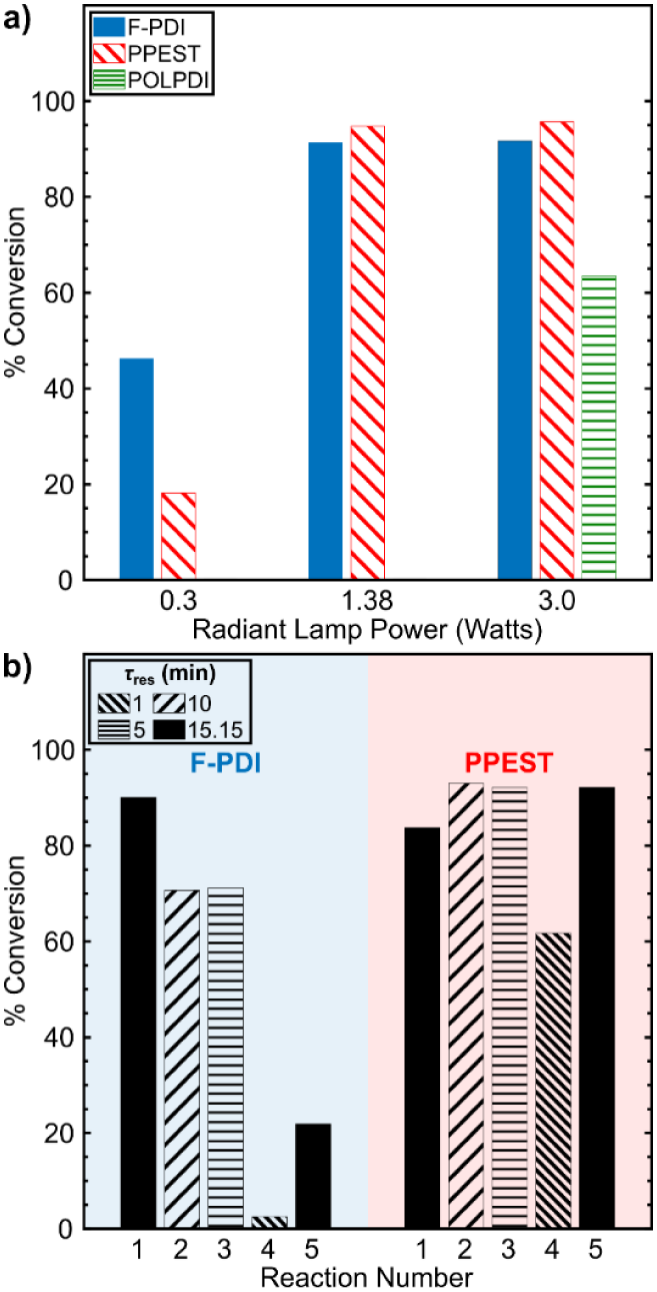
Photoconversion of **VCZ** using (a) different polymer catalysts at various radiant lamp powers and (b) **F-PDI** and **PPEST** at different residence times. Note: The reactions performed in (a) for **F-PDI** were performed with a residence time of 30.3 min.

Based on the results, **F-PDI** and **PPEST** were selected to study the effect of the residence time (and flow rate) on **VCZ** photoconversion. We note that the PFA tubing coating procedure may have some variability in polymer coating efficiency, as this process requires further optimization. Thus, freshly coated PFA tubing was prepared with **F-PDI** and **PPEST**. Because τ_res_ is dependent on the flow rate for reactors with the same volume, we reasoned that the higher flow rates needed to achieve a shorter τ_res_ could have a strong influence on coating stability. With faster flow, the friction forces of the reagent solution or solvent relative to the polymer-coated PFA tubing will be greater, possibly leading to additional loss of the catalyst.

A series of reactions were performed at maximum LED power with varied τ_res_, where the first and last reactions had the same 15.15 min τ_res_ (Figure 5b). Relative changes between the first and last reaction would allow for a qualitative understanding of the polymer coating stability. In other words, similar conversions between the first and last reaction in the series would be indicative of PC retention. With respect to **F-PDI**, the conversion achieved for the first reaction on the newly coated PFA was 90.1%, nearly the same as that for the same reaction in Figure 5a. At the lowest τ_res_ of 1 min (2 mL·min^–1^), the **VCZ** conversion was significantly lower at 2.5%. This was not unexpected since the reaction had less time to take place and would most likely lead to inefficient ***t*** **-DCZCB** formation. Lower flow rates (to give longer τ_res_) did result in higher conversions around 70%; however, the final reaction that was under the same conditions as the first did not yield a similar result. Indeed only 21.8% **VCZ** conversion was achieved, which is likely due to the loss of **F-PDI** under high flow conditions. On the other hand, as shown in Figure 5b, **PPEST** proved to be much more resistant to high flow rate conditions and successfully achieved a conversion of ∼62% even at 2 mL·min^–1^. It was therefore concluded that **PPEST** was the most robust polymer photocatalyst of the three in this study. These high conversions (<90%) are comparable with the reaction in both batch and flow systems.^27^

Additional tests were performed to gauge whether **PPEST** leached from the PFA tubing over the course of a reaction under continuous flow in acetone, the full details of which are described in the Supporting Information. The reaction of **VCZ** with PFA-bound **PPEST** was performed using 525 nm light at 0.3 W power, with a residence time of 15.15 min. An aliquot of the crude reaction product was analyzed for the presence of **PPEST** by fluorescence spectroscopy (Figure S24). As observed in the emission spectrum of the crude reaction product mixture in the presence and absence of a catalyst, no **PPEST** emission was detected, indicating that the coating was stable under these flow rate conditions. It is therefore likely that a reduction in catalyst-coating performance results from either higher flow rates, or extended use (and irradiation) over time.

To further gauge the relative stability of **PPEST** and its efficacy for continuous flow photochemistry applications, in-line ^1^H NMR spectroscopy was employed via a 60 MHz benchtop NMR spectrometer (Figure S21). Due to the lower resolution of benchtop NMR spectrometers, and the desire for single-peak proton solvents (acetone, MeCN, etc.), **VCZ** conversion was tested in pure acetone at a range of concentrations up to 50 mg·mL^–1^ (Figure S20). The reaction could be performed at 50 mg·mL^–1^ with good signal-to-noise, without precipitation of the product, and with conversions up to ∼94%. Thus, a freshly coated **PPEST**-coated PFA reactor coil was prepared and the flow path adjusted such that the product solution continuously passed through the NMR spectrometer via a flow cell. The reaction was conducted at a radiant lamp power of 3.0 W, with a 15.15 min τ_res_ (0.132 mL·min^–1^) continuously for 18 h. Figure 6 shows a diagram of the flow path incorporating the in-line NMR instrument. After an initial 5 min delay at the start of the reaction, ^1^H NMR spectra were acquired every 5 min (173 spectra in total) using the parameters described in Table S3. An open-source Python module, nmrglue,^28,29^ was used to process the NMR spectra. All of the spectra were imported and passed through a Hanning filter to ensure proper smoothing of the free induction decay (FID), thereby improving the autophasing by nmrglue. To improve the accuracy of peak integrations in the low-fidelity data, each spectrum was treated with local baseline subtraction before integration and calculation of the photoconversion. An example of the product mixture spectrum showing the local baseline subtraction and integration regions of the product (triplet, 6.53 ppm) and reagent (doublets, 5.72–5.09 ppm) is provided in Figure 6b. Full details are described in the Supporting Information.

**Figure 6:**
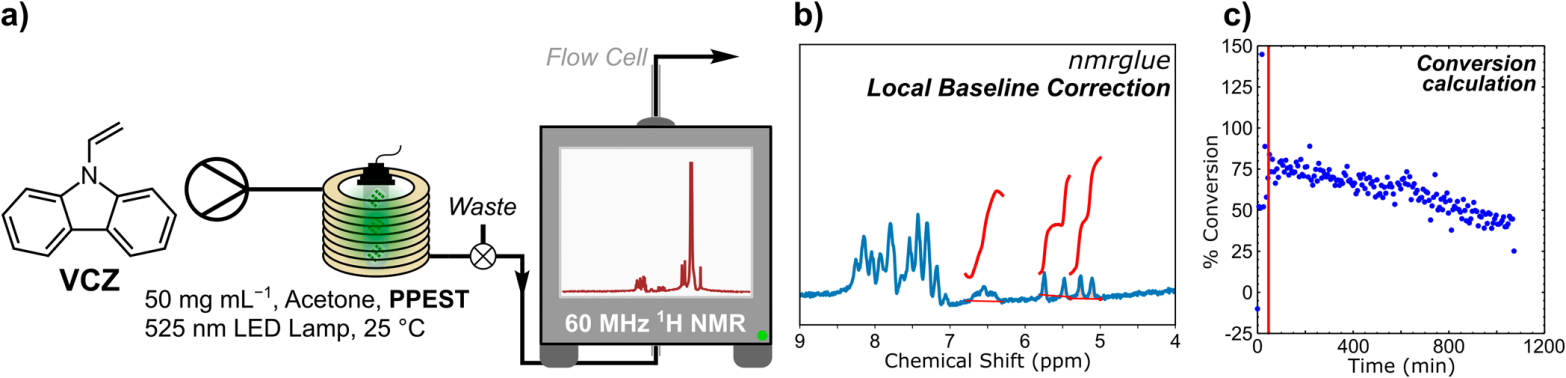
Photoconversion of **VCZ** with **PPEST** over 18 h continuous flow. (a) Flow diagram depicting in-line ^1^H NMR spectrometer reaction monitoring and a typical crude NMR spectrum. Arrows indicate the direction of the flow. (b) Magnified view of a crude reaction mixture ^1^H NMR spectrum with local baseline correction and integration of the product triplet and starting material doublets via nmrglue. (c) **VCZ** conversion data over 18 h continuous flow. The red line indicates the time after ∼3 residence times, where steady-state is commonly observed (45.45 min, 3× τ_res_). The first data point (0 min) represents the first NMR spectrum acquired following a 5 min delay time after turning the pumps on.

Figure 6c shows the conversion of **VCZ** over the course of 18 h (∼71.3 residence times) of continuous flow. As with previous experiments, approximately three reactor volumes (∼6 mL) were needed before the system reached equilibrium conditions (Figure 6, red line). While this variation would be unobservable without an in-line spectrometer, the incorporation of an in-line NMR spectrometer provided an opportunity to monitor variation and identify when steady-state conditions are achieved. Once equilibrium was reached, the conversion stabilized at around 75% for ∼100 min. Note that with the freshly coated reactor, the highest conversion was lower than that of previously prepared **PPEST** coatings. We believe that this variability is the result of some minor differences in the surface properties of PFA tubing. After reaction initialization, a steady decrease in photoconversion by ∼25–30% was observed over the course of the experiment but impressively, toward the end of 18 hours of continuous production, ∼50% conversion was still occurring. To provide an assessment of the efficiency of this continuous manufacturing method, more advanced data analysis of the in-line NMR spectra data was performed (see Supporting Information and Jupyter notebook). To ensure steady-state conditions, data acquired after 45 min was used to assess the total conversion area as a function of time. The processed data was fit to a function using Simpson’s rule, a second-order polynomial, and a third-order polynomial, all yielding very similar total areas. From the third-order polynomial expression, the percent conversion during steady-state (17 h) was 61% of the theoretical maximum. From this calculation, an estimated 4.18 g of product is produced over 17 h. Furthermore, based on the mass of **PPEST** used to coat the reactor, this equates to 0.58 g product per mg photocatalyst used. Importantly, this estimation considers the amount of catalyst used during the tubing coating process, and the amount of dye physically coated on the tubing, is considerably smaller. Therefore, it is safe to assume that the grams of product per milligram of photocatalyst are likely much higher than this estimation. Overall, the data from in-line monitoring strongly suggest that **PPEST** is a robust and reliable polymer photocatalyst for use in continuous flow.

## Conclusions

In conclusion, through the design of fluorophilic interactions, an efficient method has been developed for the functionalization of the PFA coil reactors. Specifically, these principles have been applied to fluorinated photocatalysts **F-PDI**, **PPEST**, and **POLPDI**, and their performance tested in a [2 + 2] photocycloaddition. The activity of these photocatalysts was impressive, especially considering the amount of photocatalyst was quantified to having only a few micrograms per centimeter of tubing (26.37 ± 12.79, 0.095 ± 0.039, and 0.354 ± 0.110 μg·cm^–1^, respectively). Out of the three catalysts, the **PPEST** photocatalyst proved to be the most homogeneous and robust coating leading to high conversions of **VCZ** to ***t*** **-DCZCB** with different lamp power and residence times. Additionally, **PPEST** exhibited great potential as a photocatalyst for continuous flow with high conversions (75 to ∼50%) measured by an in-line ^1^H NMR benchtop spectrometer over 18 h. Overall, these new design principles offer opportunities to create advanced catalysts and harness continuous flow chemistry for chemical synthesis.

## Supplementary Material





## Notes

Additional experimental details, materials, and methods, including photographs of experimental setup, and spectral characterization (PDF)
Experimental tabulated data from continuous monitoring reaction collected on 60 MHz nuclear magnetic resonance spectrometer along with Jupyter notebook scripts for data processing (ZIP)
