# Supplementary material for: Photoactive PFA Coating through Fluorophilic Interactions for Continuous Flow Photochemistry
Source: JACS Au. 2025 Nov 25;5(12):5965–72. doi: 10.1021/jacsau.5c00804 (PMC12728596; doi:10.1021/jacsau.5c00804)
Supplement: Supplementary file 1 [file au5c00804_si_001.pdf]

Supporting Information (SI) for:

Photoactive PFA Coating through Fluorophilic Interactions for Continuous Flow Photochemistry

Jesús Castro-Esteban,[‡,a] John H. Dunlap,[‡,b,c] Benedikt S. Schreib,[‡,a] Peter Mirau,[b] Christopher A. Crouse,[b] Timothy M. Swager*[,a] and Luke A. Baldwin*[,b]

[a]Department of Chemistry, Massachusetts Institute of Technology, Cambridge, MA 02139, USA
[b]Materials and Manufacturing Directorate, Air Force Research Laboratory, Wright-Patterson AFB, OH 45433, USA
[c]AV, Inc., Dayton, OH 45432, USA

[‡] These authors contributed equally to this work.
*Corresponding Authors: tswager@mit.edu, luke.baldwin.1@us.af.mil

1. **Materials and Instruments S2**
2. **Synthesis of F-PDI, Monomers, and Polymers S2**
3. **Gel Permeation Chromatography S4**
4. **UV-vis Spectra (Molar Absorptivity) S5**
5. **PFA Tubing Functionalization S6**
6. **Photocatalyst Quantification and Leaching Studies S8**
7. **Continuous Flow Photoreactions S10**
8. **Continuous Flow with In-Line ^{1}H NMR Spectroscopy S13**
9. **Continuous Flow Catalyst Leaching Experiments S18**
10. **NMR Spectra S20**
11. **Alternative Reactions Studied S23**
12. **References S28**

1. Materials and Instruments

All reactions were carried out under argon using oven-dried glassware. Flash column chromatography was carried out using silica gel (high-purity grade, pore size 60 Å, 230-400 mesh particle size, Sigma Aldrich). Commercial reagents and solvents were purchased from Sigma Aldrich, SynQuest, Alfa Aesar, Ambeed or VWR, and were used without further purification unless otherwise noted below.

^{1}H, ^{19}F and ^{13}C NMR spectra were recorded using either a four-channel Bruker Avance Neo spectrometer operating at 600.14 MHz, or a Bruker Avance 400 MHz spectrometer. High resolution mass spectra (HRMS) were obtained using a JEOL AccuTOF 4G equipped with an ionSense direct-analysis-in-real-time (DART) source. Molecular weight and dispersity of the polymers was obtained using an Agilent 1260 Infinity system equipped with an Agilent PLgel guard column (5 µm; 50 x 7.5 mm) and three Agilent PLgel analytical columns (5 µm; 300 x 7.5 mm; 105, 104, and 103 Å pore sizes). The instrument was calibrated with polystyrene standards between 1.7 and 3150 kg mol^{-1}. All samples were prepared in HPLC-grade tetrahydrofuran. Each run was performed at a 1.0 $mLmin^{-1}$ flow rate and 35 °C. Molecular weight values were calculated using ChemStation GPC Data Analysis Software (rev. B.01.01) based on the refractive index signal.

UV-vis absorption spectra were recorded on a Cary 5000 spectrophotometer and corrected for background signal with a solvent-filled cuvette for solutions and glass slide for thin films. Fluorescence measurements were performed using a Horiba Quanta-ϕ fluorescence spectrophotometer, using right-angle detection for solutions, right-angle detection with an optical fiber for tubing, and front-face detection for thin films.

Perfluoroalkoxy (PFA) tubing (1.6 mm I.D., 3.175 mm O.D.) for coating experiments was purchased from Zeus. PFA tubing (2 mL volume, 1.3 mm I.D., 1.6 mm O.D.), LED lamps (440 nm and 525 nm wavelengths), and other flow chemistry equipment were purchased from Vapourtec Ltd.

2. Synthesis of F-PFI, Monomers, and Polymers

F-PDI[1] and pentiptycene diacetylene **1**[2] were prepared following published procedures (Figure S1).

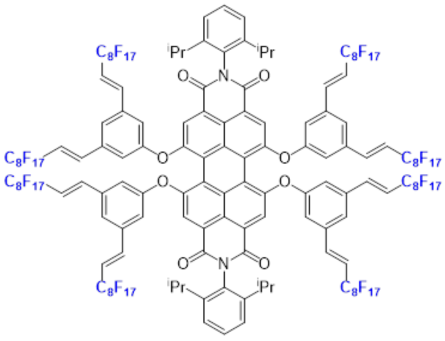

F-PDI

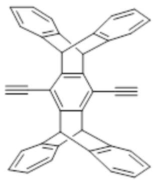

1

Figure S1. Compounds previously reported.

*Synthesis of fluorinated dibromo ester **4**:*

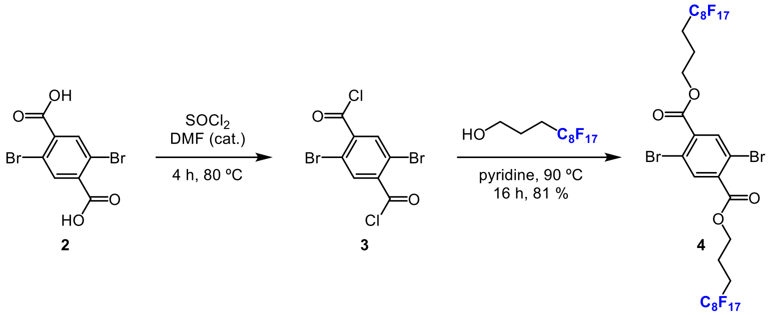

A mixture of 2,5-Dibromoterephthalic acid (**2**, 648 mg, 2.0 mmol), $SOCl_2$ (2.9 mL, 40 mmol) and a catalytic amount of anhydrous DMF (2 drops) was stirred at 80 °C (reflux) for 4 h under argon atmosphere. The excess of $SOCl_2$ was removed by distillation and the crude product was further dried under vacuum for 2 h to afford a white solid. Subsequently, the crude product was dissolved in pyridine (5 mL), 3-(perfluorooctyl)propanol (2.10 g, 4.4 mmol) was added, and the mixture was stirred under argon atmosphere at 90 ºC for 16 h. Next day, the solvent was evaporated under reduced pressure and the crude product was dissolved in a hot mixture of $CHCl_3$:EtOH (1:1). The precipitate after cooling down was filtered and rinsed with EtOH to afford the fluorinated diester **4** (2.01 g, 81 %) as a white solid.
^{1}H NMR (600 MHz, $CDCl_3$) δ: 8.02 (s, 2H), 4.45 (t, J = 6.2 Hz, 4H), 2.37 – 2.21 (m, 4H), 2.18 – 2.07 (m, 4H) ppm. **^{19}F NMR** (565 MHz, $CDCl_3$) δ: -80.82, -114.31, -121.67, -121.92, -122.73, -123.43, -126.14 ppm. **^{13}C NMR** (151 MHz, $CDCl_3$) δ: 164.26 (2xC), 136.75 (2xCH), 135.79 (2xC), 120.20 (2xC), 118.30 (2xC), 117.28 (2xC), 111.29 (2xC), 111.16 (2xC), 110.94 (2xC), 110.88 (2xC), 110.37 (2xC), 108.54 (2xC), 65.00 ($2xCH_2$), 28.16 ($2xCH_2$), 20.12 ($2xCH_2$) ppm. **HRMS** (DART) *m/z* calculated for $C_{30}H_{15}Br_2F_{34}O_4$ $[M+H]^+$, 1242.87887; found, 1242.87703.

General procedure for Sonogashira polymerization:

A degassed solution of diisopropylamine/toluene (2:3, 0.015 M) was added to a Schlenk flask containing monomers, copper (I) iodide, and tetrakis(triphenylphosphine)palladium (0). The flask was deoxygenated by three freeze-pump-thaw cycles and flushed with argon. The reaction mixture was stirred at 80 °C for 72 hours. After cooling down to r.t., the polymer precipitates in the solvent mixture. Then, tetrahydrofuran was added to dissolve the polymer which was then precipitated into methanol twice and washed with hot methanol and acetone.

*Synthesis of **PPEST**:*

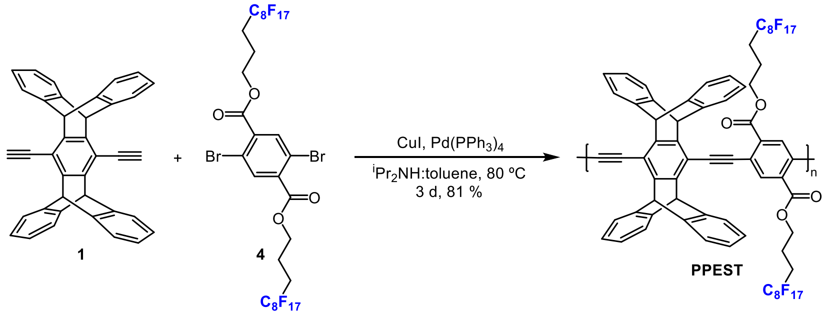

PPEST was prepared using dialkyne pentiptycene **1** (41 mg, 0.084 mmol), dibromo ester **4** (104.5 mg, 0.084 mmol), CuI (0.96 mg, 0.00504 mmol) and $Pd(PPh_3)_4$ (11.6 mg, 0.0101 mmol) to afford the polymer as a green solid (81 %).

^{1}H NMR (600 MHz, $CDCl_3$) δ: 8.64 (s, 2H), 7.72 – 7.38 (m, 8H), 7.14 – 7.68 (m, 8H), 6.33 – 6.13 (m, 4H), 4.91 – 4.63 (m, 4H), 2.46 – 2.29 (m, 8H) ppm. **^{19}F NMR** (565 MHz, $CDCl_3$) δ: -80.82, -114.03, -121.61, -121.86, -121.93, -122.74, -123.07, -126.14 ppm. **GPC** (THF): M_n = 26.5 kDa, $Đ$ = 2.12.
*Synthesis of **POLPDI**:*

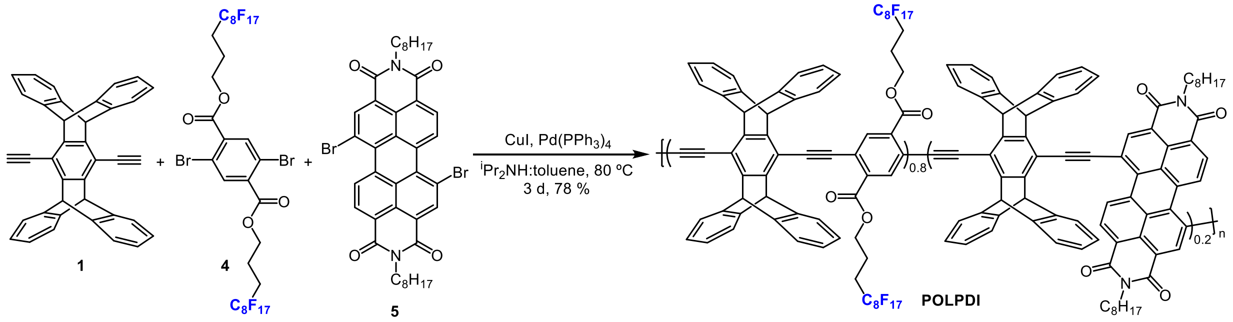

POLPDI was prepared using dialkyne pentiptycene **1** (97.65 mg, 0.200 mmol), dibromo ester **4** (199.1 mg, 0.160 mmol), dibromoPDI **5** (30.9 mg, 0.040 mmol), CuI (2.28 mg, 0.012 mmol) and $Pd(PPh_3)_4$ (27.75 mg, 0.024 mmol) to afford the polymer as a dark red solid (78 %).
^{1}H NMR (600 MHz, $CDCl_3$) δ: 10.41 (d, J = 9.0 Hz, 0.38H), 9.47 – 9.24 (m, 0.41H), 9.01 (d, J = 8.7 Hz, 0.38H), 8.78 – 8.55 (m, 1.48H), 7.67 – 7.36 (m, 9H), 7.27 – 6.88 (m, 8H), 6.46 – 5.99 (m, 4H), 4.80 (t, J = 6.4 Hz, 3.2H), 4.40 (s, 0.86H), 2.52 – 2.21 (m, 7H), 1.93 (s, 1H), 1.50 – 1.20 (m, 5.48H), 1.05 – 0.80 (m, 2H) ppm. **^{19}F NMR** (565 MHz, $CDCl_3$) δ: -80.82, -114.02, -121.61, -121.86, -121.93, -122.74, -123.07, -126.14 ppm. **GPC** (THF): M_n = 12.8 kDa, $Đ$ = 2.03.

3. Gel Permeation Chromatography

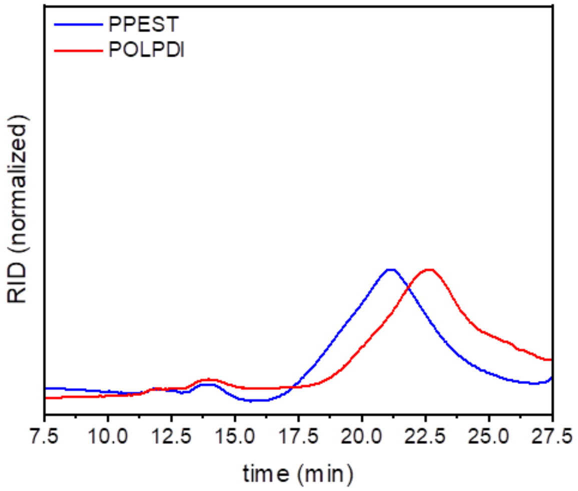

Figure S2. GPC traces of **PPEST** and **POLPDI**.

4. UV-vis Spectra (Molar absorptivity)

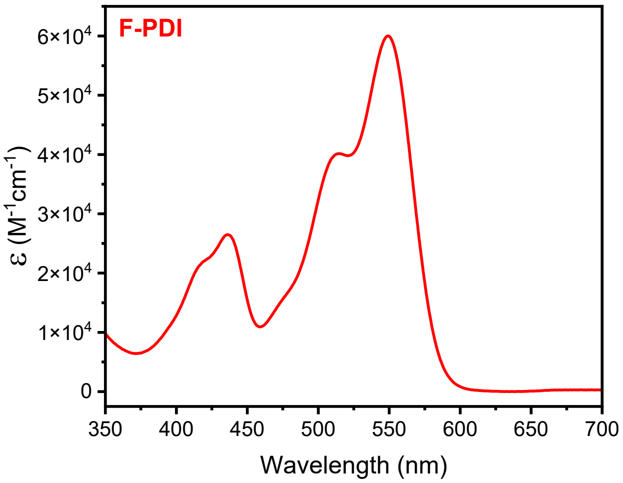

Figure S3. Molar absorptivity of **F-PDI** in benzotrifluoride.

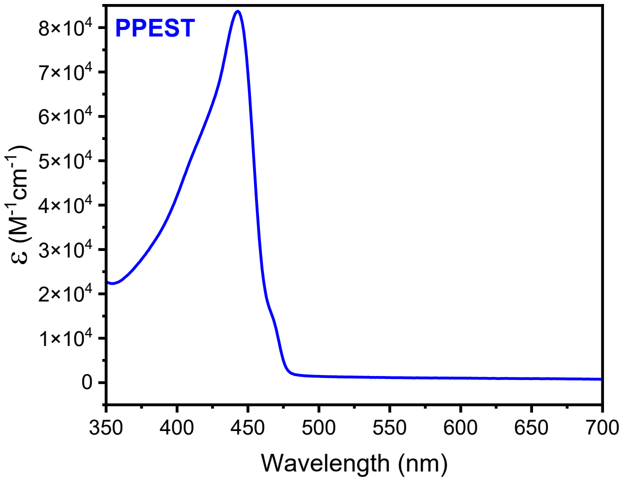

Figure S4. Molar absorptivity of **PPEST** in benzotrifluoride.

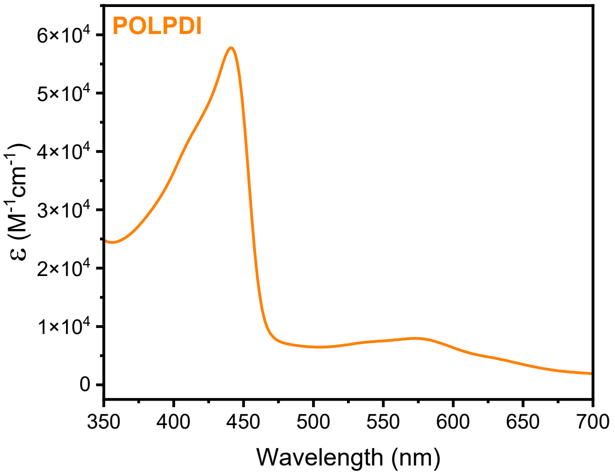

Figure S5. Molar absorptivity of **POLPDI** in benzotrifluoride.

5. PFA Tubing Functionalization

Coating methodology: First, **F-PDI** and polymer solutions were prepared in anisole at 100 °C (5 mg mL^{-1} for the **F-PDI** and 2.5 mg mL^{-1} for the polymers). The PFA tubing was preheated for 1 hour at 120 °C in a silicone oil bath, then the hot polymer solution was slowly injected until the entire tube was filled and free of air bubbles. After 10 minutes the tubing was allowed to cool to r.t. over 1 hour. The solution was drained and the tube flushed with 10 mL MeCN to remove residual photocatalyst solution and unbound dye. Finally, the tubing exterior was washed with hexanes to remove any remaining oil, then dried in a vacuum chamber at r.t. overnight. Drying afforded **F-PDI** and polymer-coated PFA tube reactors as shown in Figure S6 and stored in the dark until use. Prior to continuous flow experiments, the tubing was carefully coiled around a 2 mL reactor cartridge (Vapourtec).

After each reaction or test, the catalyst-modified tubing was rinsed with MeCN, drained of solvent, and stored dry in the dark at room temperature. No photocatalyst degradation, or lower reactivity efficiency was noted following these steps.

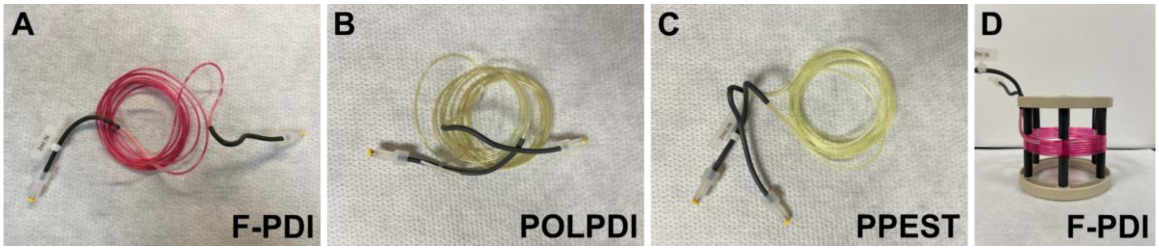

Figure S6. a) **F-PDI**-functionalized PFA coil reactor.; b) **PPEST**-functionalized PFA coil reactor.; c) **POLPDI**-functionalized PFA coil reactor.

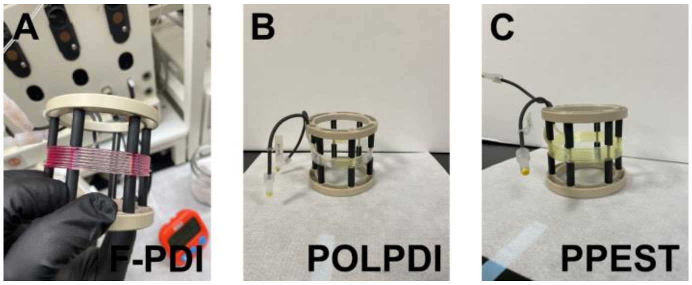

Figure S7. Representative coated PFA tubing after running test reactions with **VCZ**.

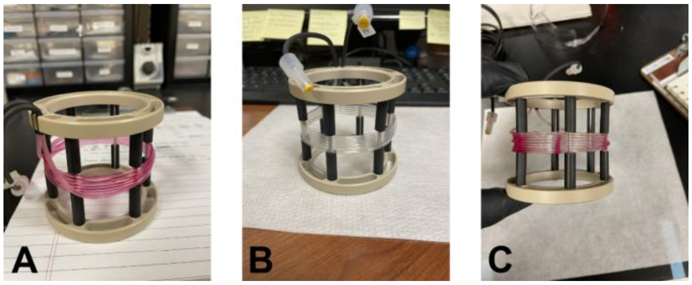

Figure S8. Test of ability to re-coat used PFA tubing with **F-PDI**. (A) New **F-PDI**-functionalized PFA tubing, (B) Reactor used for [2+2] photocycloaddition reactions then coating removed with acetone, and (C) The same reactor after attempting to re-functionalize the PFA with **F-PDI**. The final image shows that PFA tubing that has been previously used for photochemistry cannot be re-functionalized with **F-PDI** to achieve a homogeneous catalyst coating. **F-PDI** adhered to PFA only in places where it was not directly irradiated by the LED lamp.

In addition, PFA tubing was functionalized with a non fluorous version of PDI to demonstrate the importance of the perfluoroalkyl chains for efficient fluorophilic interactions (Figure S9).

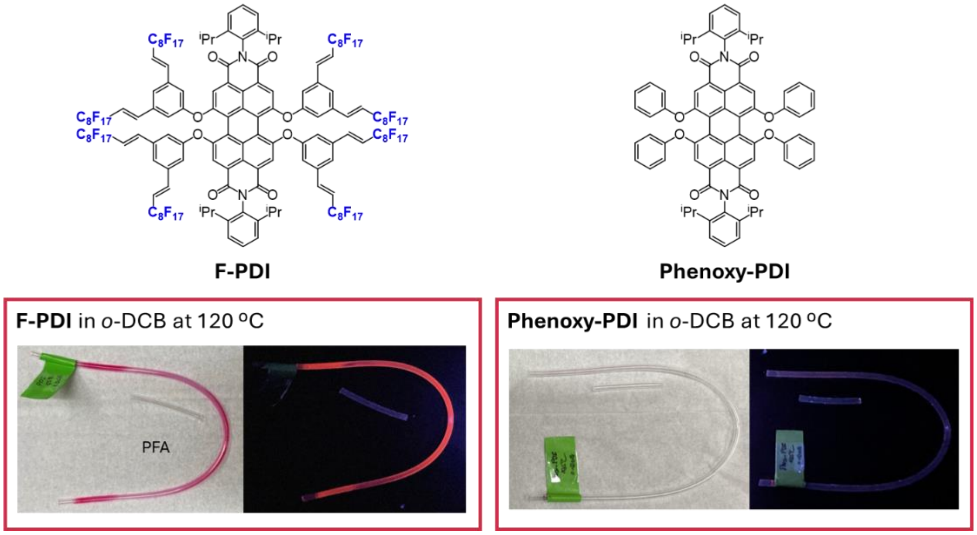

Figure S9. PFA tubing after functionalization with a solution of **F-PDI** in *o*-DBC and the comparation with a non fluorous PDI version (**Phenoxy-PDI**).

Extension of the coating methodology to other fluoropolymers such as PTFE:

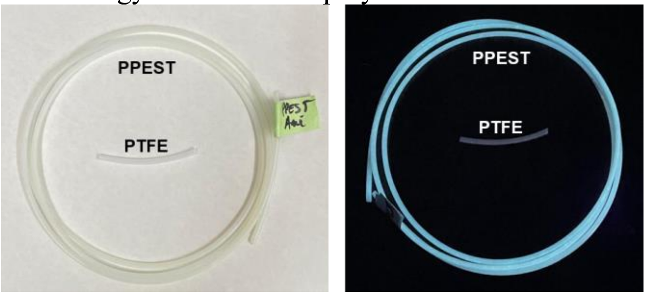

Figure S10. PTFE tubing after functionalization with a solution of **PPEST**.

6. Photocatalyst Quantification and Leaching Studies

Photocatalyst quantification studies were performed under static conditions by immersing 3-5 cm pieces of coated tubing in 3-4 mL of solvent for 30 minutes to promote coating-solvents interactions. The absorption of solutions in benzotrifluoride was measured in quartz cuvettes (3 mL) and the mass of each photocatalyst was determined by using the Beer-Lambert law equation. Five replicates were measured to obtain standard deviations from three different PFA coil reactors with 1, 2, and 4 mL.

Table S1. Photocatalyst quantification using benzotrifluoride solutions. Partial quantification for **PPEST** and **POLPDI** since part of the dye remains coated to the tubing.

	1 mL coil	2 mL coil	4 mL coil	Average
**F-PDI** (ε_{549} = 60,019 $M^{-1}cm^{-1}$)	10.01± 1.85 µg·cm^{-1}	27.85± 1.39 µg·cm^{-1}	41.24± 5.39 µg·cm^{-1}	26.37± 12.79 µg·cm^{-1}
**PPEST** (ε_{443} = 83,700 $M^{-1}cm^{-1}$)	0.078± 0.008 µg·cm^{-1}	0.058± 0.012 µg·cm^{-1}	0.149± 0.030 µg·cm^{-1}	0.095± 0.039 µg·cm^{-1}
**POLPDI** (ε_{441} = 57,789 $M^{-1}cm^{-1}$)	0.229± 0.024 µg·cm^{-1}	0.337± 0.035 µg·cm^{-1}	0.497± 0.097 µg·cm^{-1}	0.354± 0.110 µg·cm^{-1}

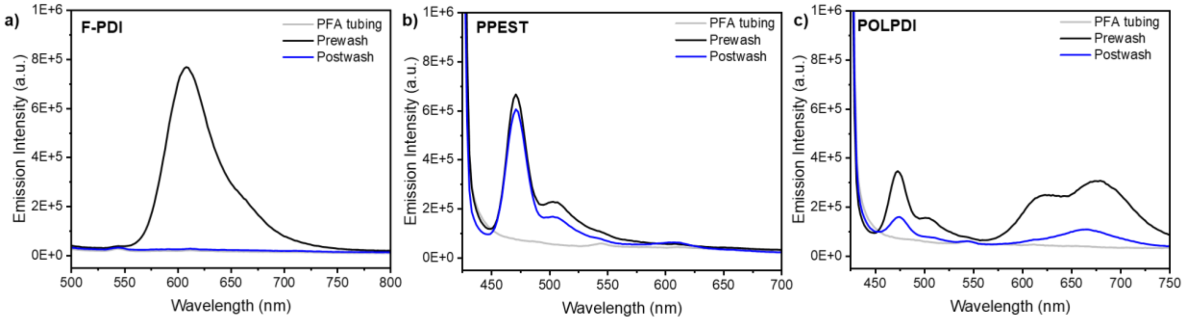

Figure S11. Emission spectra of PFA tubing, and dye-coated PFA tubing before and after benzotrifluoride.

Dye leaching studies for **F-PDI**, **PPEST** and **POLPDI** using common organic solvents (e.g., Acetone, MeOH, Toluene), reaction mixture conditions (Acetone/MeCN, 1/5) and solvents in which the dyes are soluble (e.g., Benzotrifluoride). Studies performed under static conditions by immersing 3 cm of coated tubing pieces in the solvents for 30 minutes to promote coating-solvents interactions. Fluorescence was used for a qualitative analysis of each solution because it is more sensitive than absorbance at low concentrations, where small dye amounts fall below the UV-vis detection threshold.

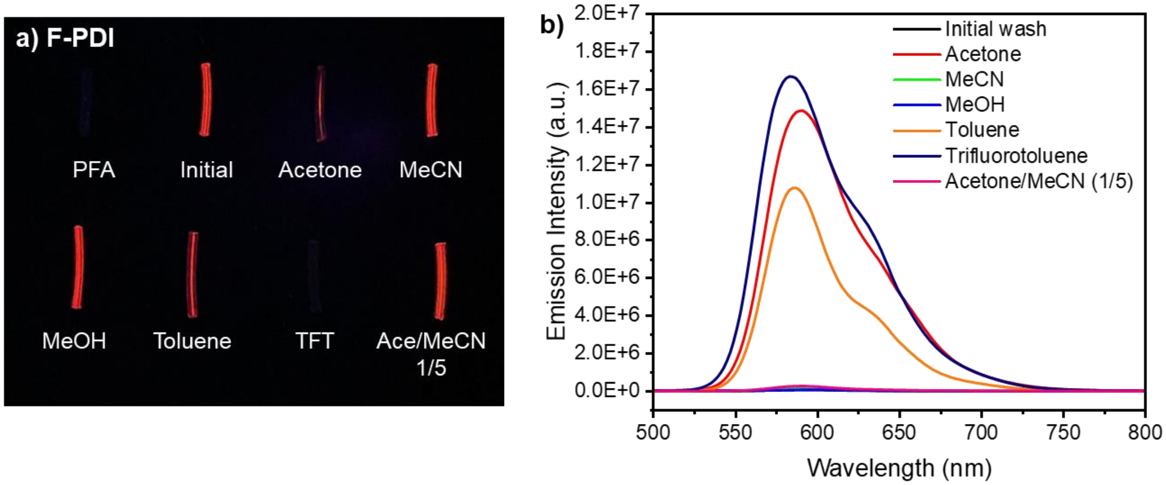

Figure S12. Pictures of the tubing after leaching experiments and emission spectra for **F-PDI**.

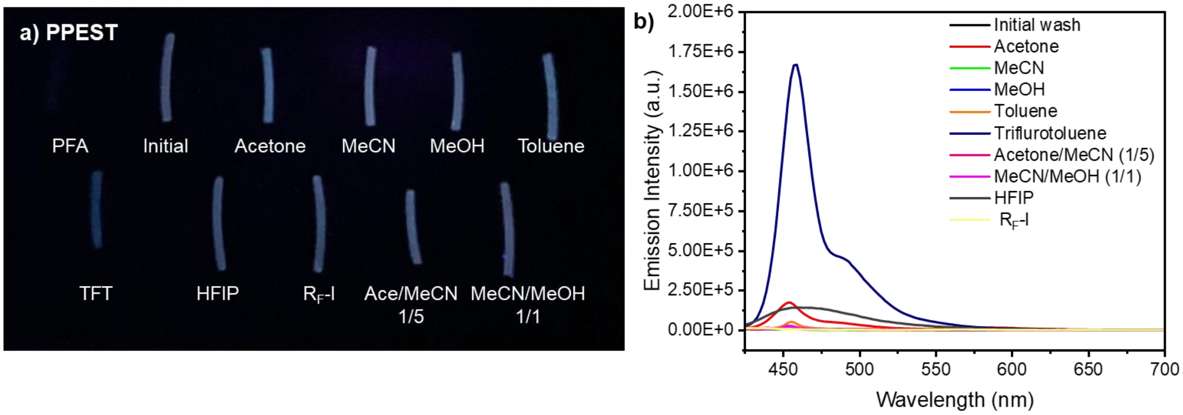

Figure S13. Pictures of the tubing after leaching experiments and emission spectra for **PPEST**.

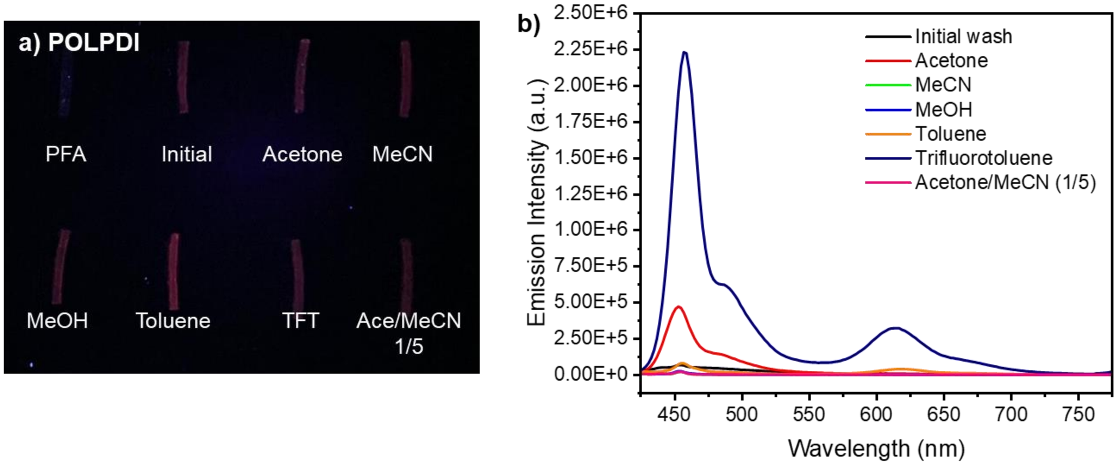

Figure S14. Pictures of the tubing after leaching experiments and emission spectra for **POLPDI**.

Additional photobleaching studies for **PPEST** in THF solutions under ambient conditions (air) and argon atmosphere. Figure S15 shows the absorption monitoring of both solutions under 525 nm irradiation for 18 hours.

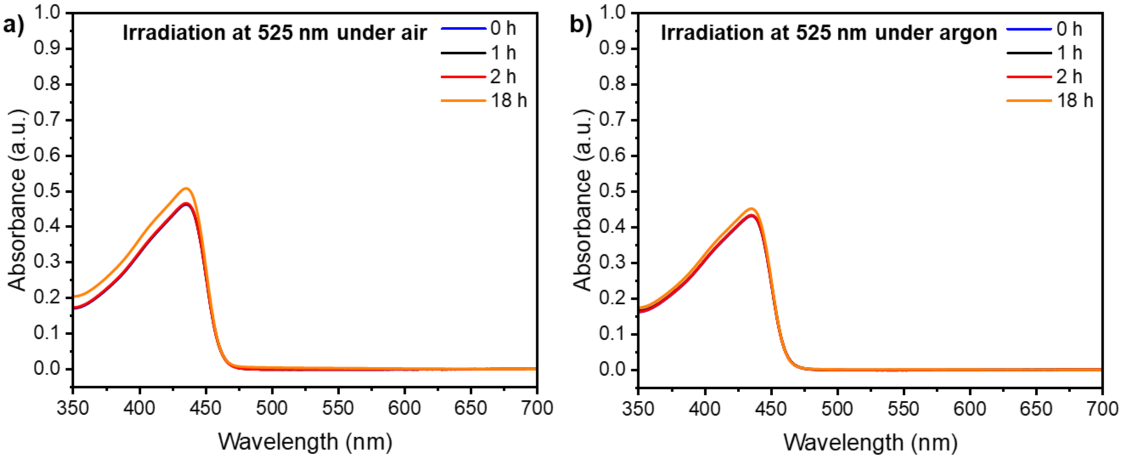

Figure S15. Photobleaching studies for **PPEST** solutions in THF under air and argon atmosphere.

7. Continuous Flow Photoreactions

Continuous flow reactions were performed using a Vapourtec R-Series modular flow chemistry system with acid resistant R2 C+ piston pumps. Reactions were conducted using 2 mL PFA tube reactors (1.3 mm I.D., 1.6 mm O.D.) in a Vapourtec UV-150 photochemical reactor equipped with LED lamps (440 nm and 525 nm) (Figure S16). Reactions were conducted at 25 °C without a back pressure regulator, and the temperature was monitored and controlled via the Vapourtec R-Series system. Aliquots of the product mixture were collected under steady-state conditions using a Gilson GX-241 Fraction Collector. The system was thoroughly rinsed with solvent before use.

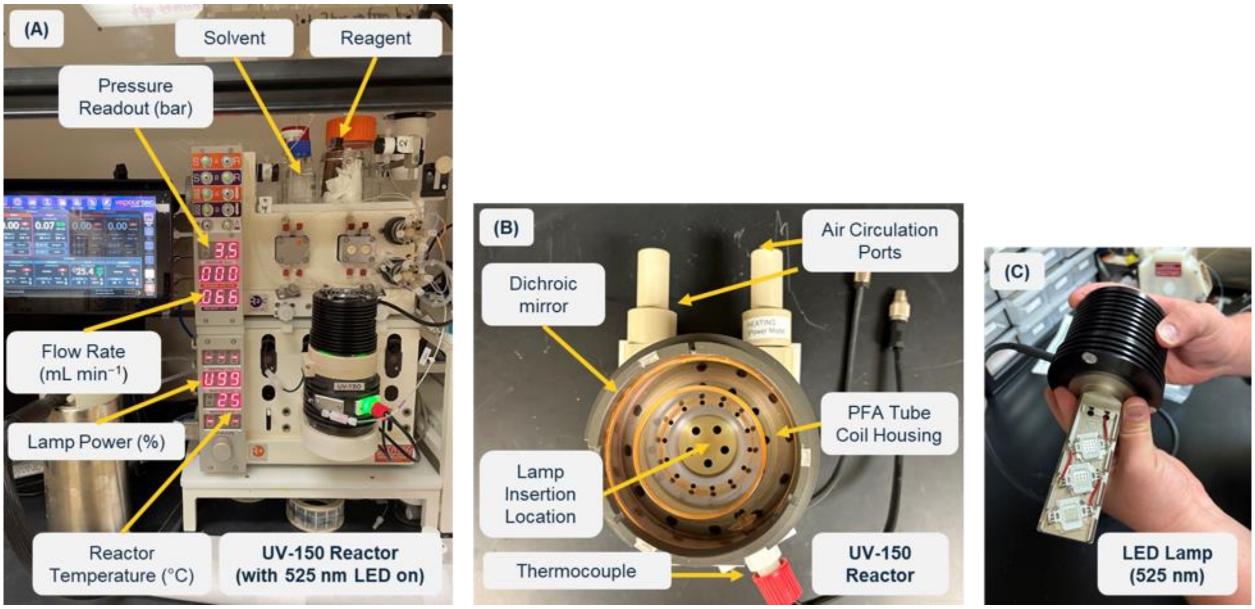

Figure S16. Vapourtec R-Series Flow System with UV-150 Photochemical Reactor. (A) UV-150 Reactor setup for reactions with 525 nm LED at maximum power. (B) Image of the UV-150 Reactor components. (C) Vapourtec 525 nm LED lamp.

For a given reaction, the desired combination of LED lamp and polymer-coated tube were setup in the UV-150 reactor. All reactions were conducted at 25 °C without a back pressure regulator. The residence time (τ_{res}) and lamp power (%) for each reaction was set using the R-Series software. For experiments performed at different **VCZ** concentrations, separate solutions were prepared for individual reactions. A schematic showing the flow chemistry setup is shown in Figure 4 of the main text.

The % conversion of **VCZ** to ***t*-DCZCB** was calculated using the ^{1}H NMR integrations for the product cyclobutane protons alpha to the pyrrole N (*t*, 6.28 ppm, 2H) and **VCZ** downfield vinyl protons (*d*, 5.54, 1H ppm) using Equation 1 below. Note that minor shifts in the peak positions may be observed for in-line NMR experiments, where a low-field NMR was used and measurements were done in acetone. In this case, the pyrrole N triplet of ***t*-DCZCB** and downfield **VCZ** vinyl proton doublet were observed at 6.48 ppm and 5.8 – 5.4 ppm, respectively.

$$\% \text{ Conversion} = \left[\frac{\frac{\int \text{t}DCZCB}{\#H_1}}{\frac{\int \text{t}DCZCB}{\#H_1}+\frac{\int VCZ}{2\times\#H_b}}\right]\times 100 = \left[\frac{\frac{\int \text{t}DCZCB}{2}}{\frac{\int \text{t}DCZCB}{2}+\frac{\int VCZ}{2}}\right]\times 100 \qquad \text{Equation (1)}$$

An isolated yield of the [2+2] cycloaddition product, ***t*-DCZCB**, was obtained for the reaction of **VCZ** with **PPEST** under 525 nm light, 3.0 Watt power, and 15.15 min τ_{res}. The collection volume was increased to allow for the collection of additional product (5 mL, nominally 250 mg ***t*-DCZCB** theoretical yield). Two replicate reactions were performed on a new **PPEST**-coated PFA reactor that had not been used previously.

The first reaction was used to obtain an isolated yield as follows: The crude reaction mixture was concentrated and purified by column chromatography on silica gel. The starting material eluted in hexanes after approximately two column volumes (144 mg), after which a 99:1 hexanes:ethyl acetate mixture was used to elute the product (98 mg, 39% yield).

To obtain an internal standard yield from the second reaction via qNMR, 1,3,5-trimethoxybenzene (TMB, 0.61 mmol) was added to the crude reaction mixture to determine the amount of unreacted **VCZ** (167.1 mg) and product ***t*-DCZCB** (85.2 mg, 34% yield).

Table S2. Comparison of conversion and yields for **VCZ-PPEST** photoreaction with 525 nm irradiation.

% Conversion	Isolated Yield (by mass)	Internal Standard Yield (by mass)
34 %	39 %	34%

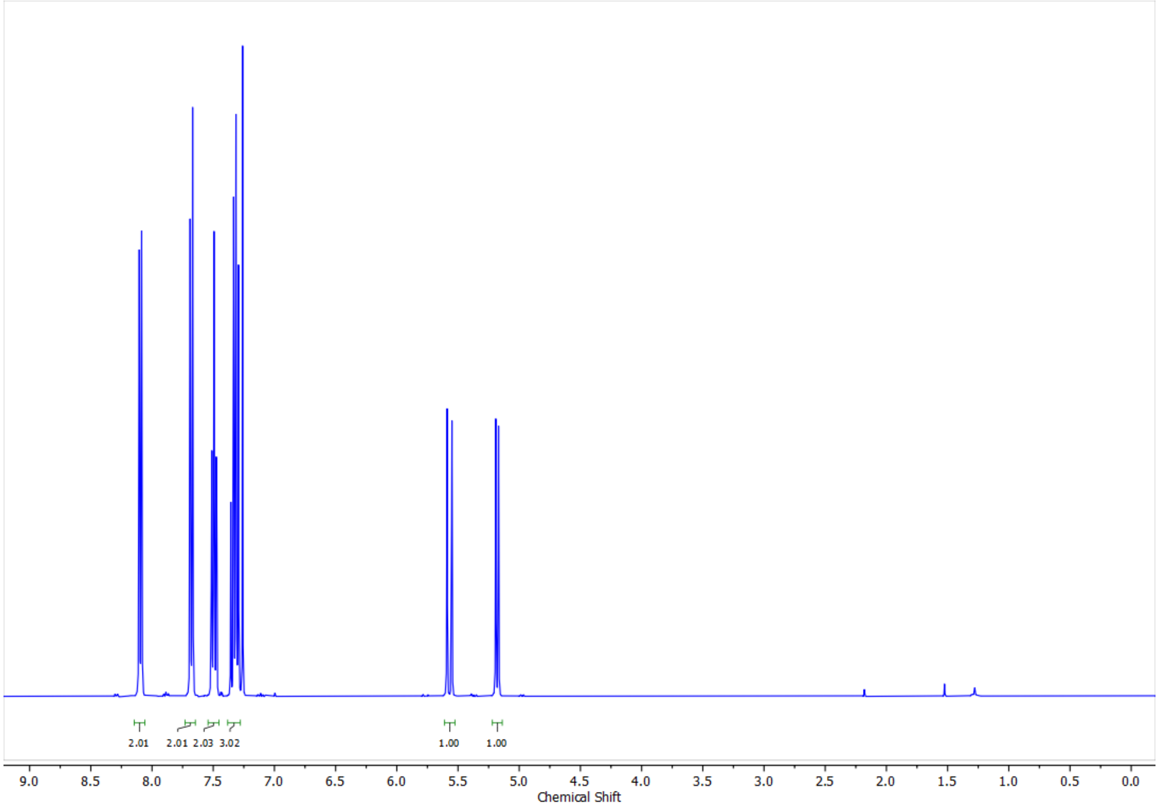

Figure S17. 400 MHz ^{1}H NMR spectrum of unreacted **VCZ** in $CDCl_3$ obtained from reaction mixture purification via column chromatography.

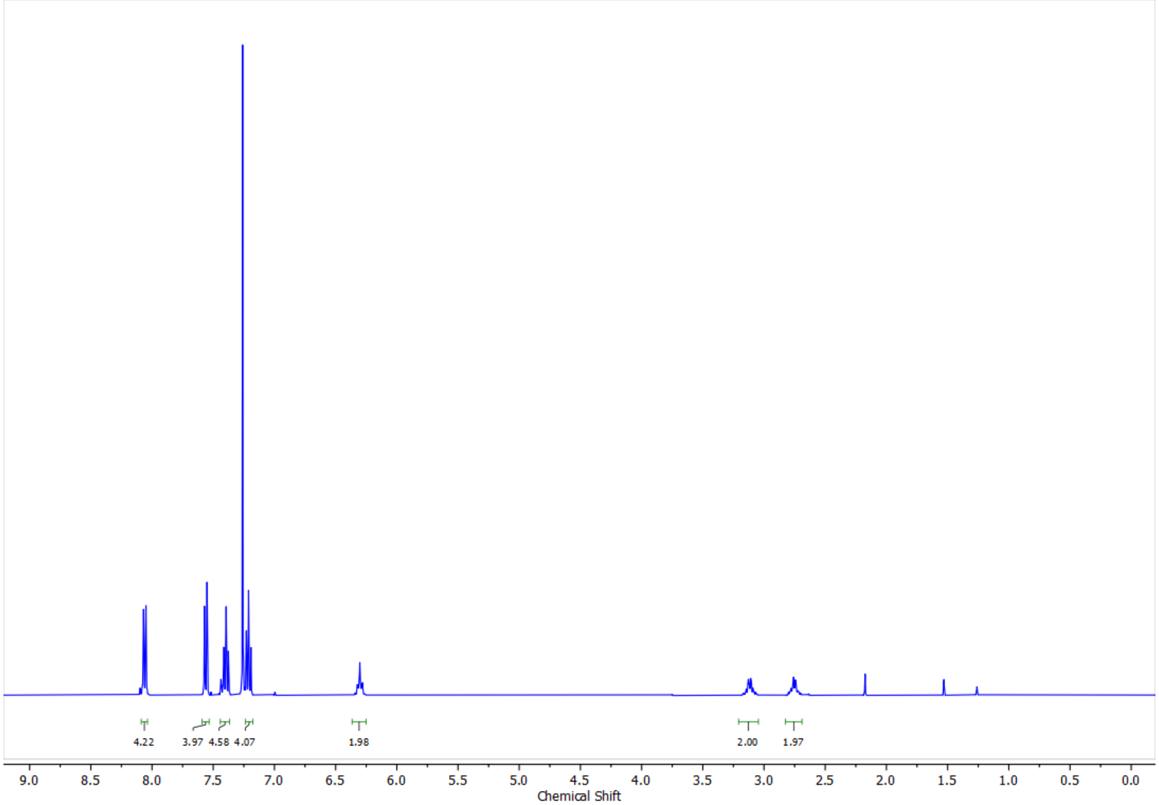

Figure S18. 400 MHz ^{1}H NMR spectrum of purified and isolated ***t*-DCZCB** product in $CDCl_3$.

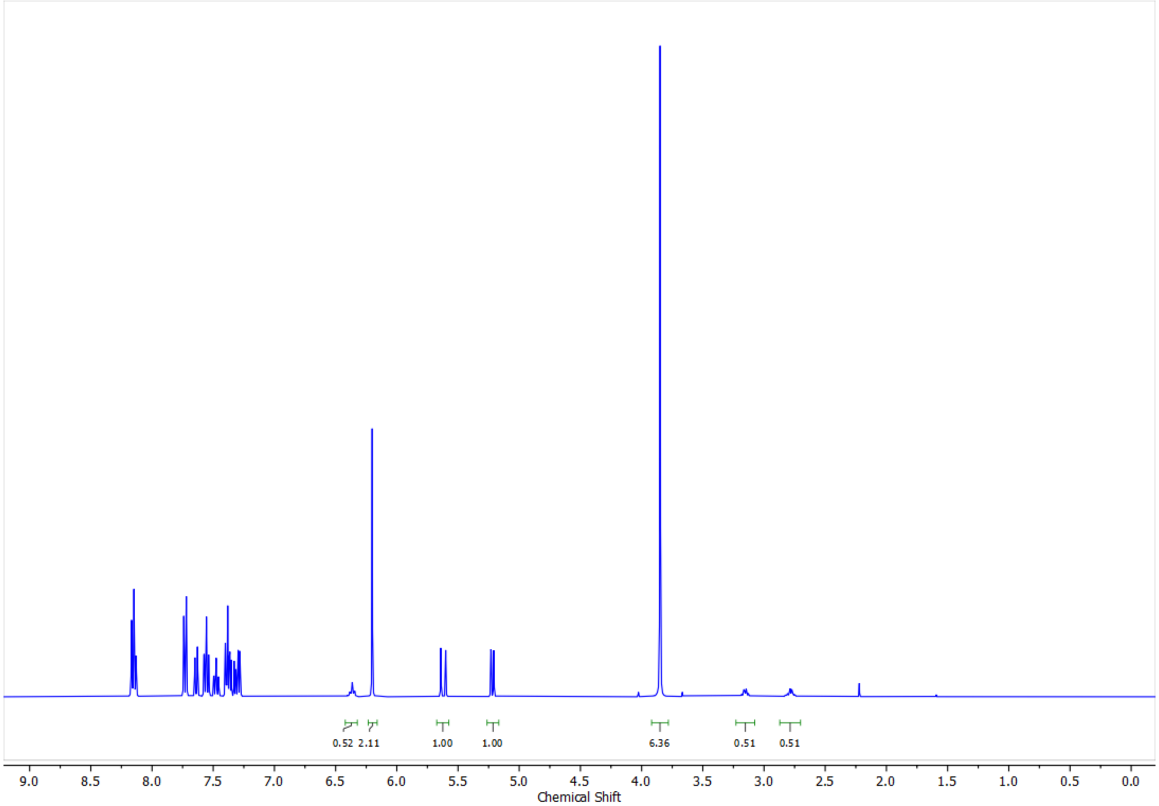

Figure S19. 400 MHz ^1H NMR spectrum of crude product mixture in $CDCl_3$ with 1,3,5-trimethoxybenzene (TMB) as internal standard. TMB peaks appear at 6.20 ppm and 3.85 ppm.

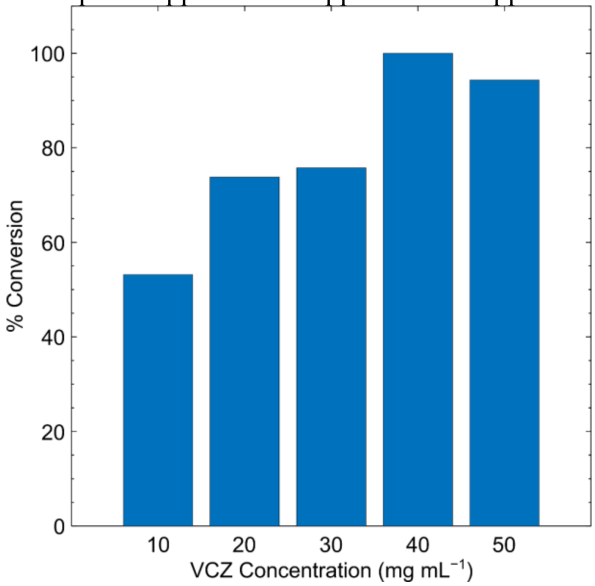

Figure S20. Photoconversion of **VCZ** in acetone over a range of concentrations. Reactions were performed with 15.15 min τ_{res} at 3.0 Watts radiant lamp power with the 525 nm LED.

8. Continuous Flow with In-Line ^1H NMR Spectroscopy

To study the stability of the photocatalyst coating over time, the flow setup was modified to incorporate in-line ^1H NMR using a Nanalysis NMReady-60pro 60 MHz benchtop spectrometer (Figure 6 of the main text). The reaction of **VCZ** (50 mg·mL^{-1}) with **PPEST** was conducted using 525 nm LED at maximum

power in acetone. The τ_{res} was set to 15.15 min (0.132 mL·min^{-1}), and the reaction run continuously for 18 hours at 25 °C. To ensure all NMR spectra were collected under steady-state conditions, three residence volumes of the reaction were diverted to waste. NMR spectra were acquired every 5 minutes of flow using the parameters described in Table S3. Raw NMR data was analyzed via the open-source Python module nmrglue, an automated processing script.[3] Prior to using nmrglue to process the spectra, they were passed through a Hanning filter to ensure proper smoothing of the free induction decay (FID). Nmrglue was then used to phase the spectra, and a local baseline subtraction was then applied. Finally, spectra were calibrated to the solvent peak (acetone) and peaks of interest were integrated for **VCZ** conversion calculation.

A more in-depth data processing script is provided in the supporting Jupyter notebook document.

After processing, a tabular overview of the results is provided as a supplemental csv file, which contains the elapsed time in the experiment for each NMR spectrum acquired (including an ~1.2 min acquisition time), as well as integral values for the product (***t*-DCZCB**) and starting material (**VCZ**). The integral value for the cyclobutane protons alpha to the pyrrole N (*t*, 6.48 ppm) are listed under "Product", while the vinyl protons of **VCZ** are listed under "Reactant". We distinguish between the downfield and upfield doublet integrations as "Reactant1" (*d*, 5.8 – 5.4 ppm) and "Reactant2" (*d*, 5.3 – 5.0 ppm), respectively. The % conversion was calculated solely based on "Product" and "Reactant" data.

To compare the conversion of **VCZ** at the end of the reaction, a 6 mL aliquot of the product solution was collected during the last hour of the experiment, concentrated by rotary evaporation to a light green-yellow oil, and ^{1}H NMR (Bruker Avance 400 MHz) measurements were recorded in $CDCl_3$.

Table S3. In-line 60 MHz NMR spectra acquisition parameters.

Spectrum Width	20 ppm
Spectrum Center	6 ppm
Scan Delay	0.5 s
Number of Points (per spectrum)	4096
Number of Scans	16
Receiver Gain	12.00 dB

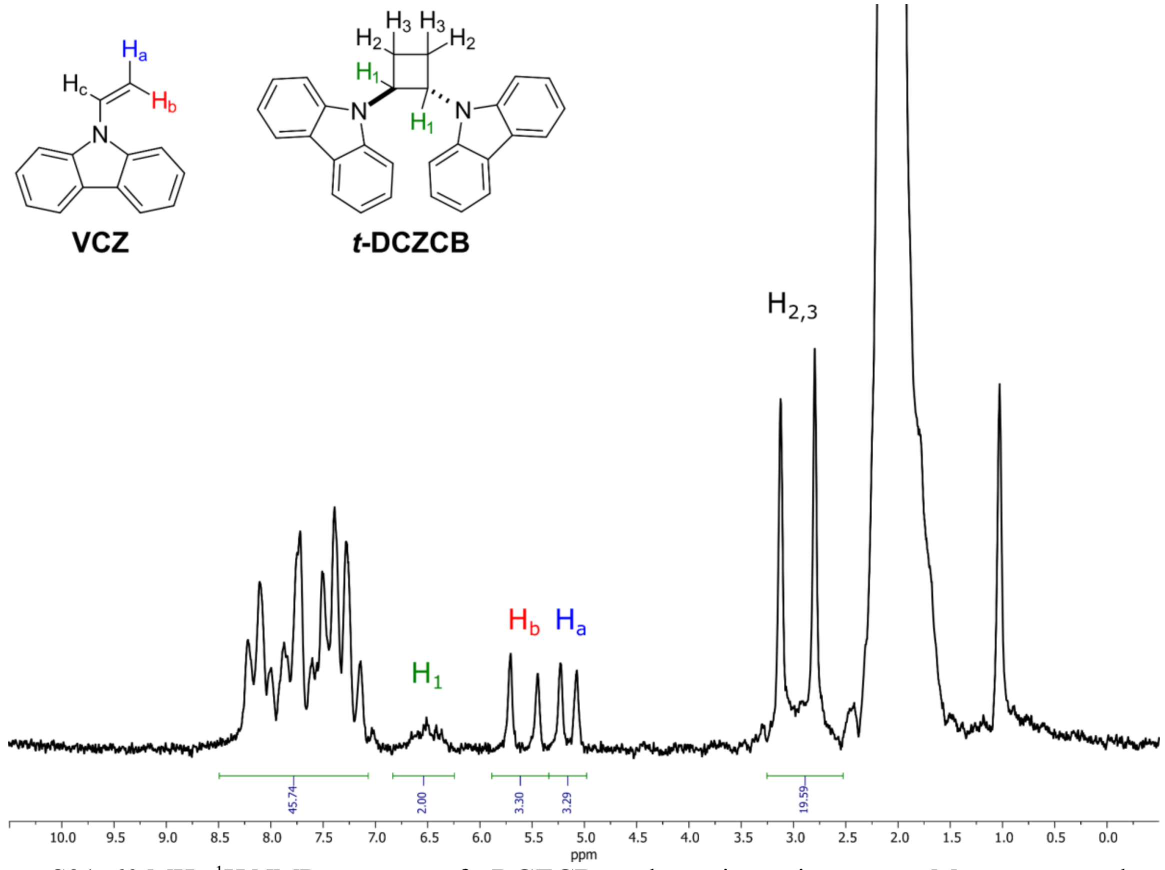

Figure S21. 60 MHz ^{1}H NMR spectrum of ***t*-DCZCB** product mixture in acetone. Measurement taken as the final measurement after 18 hours continuous flow via in-line NMR spectroscopy.

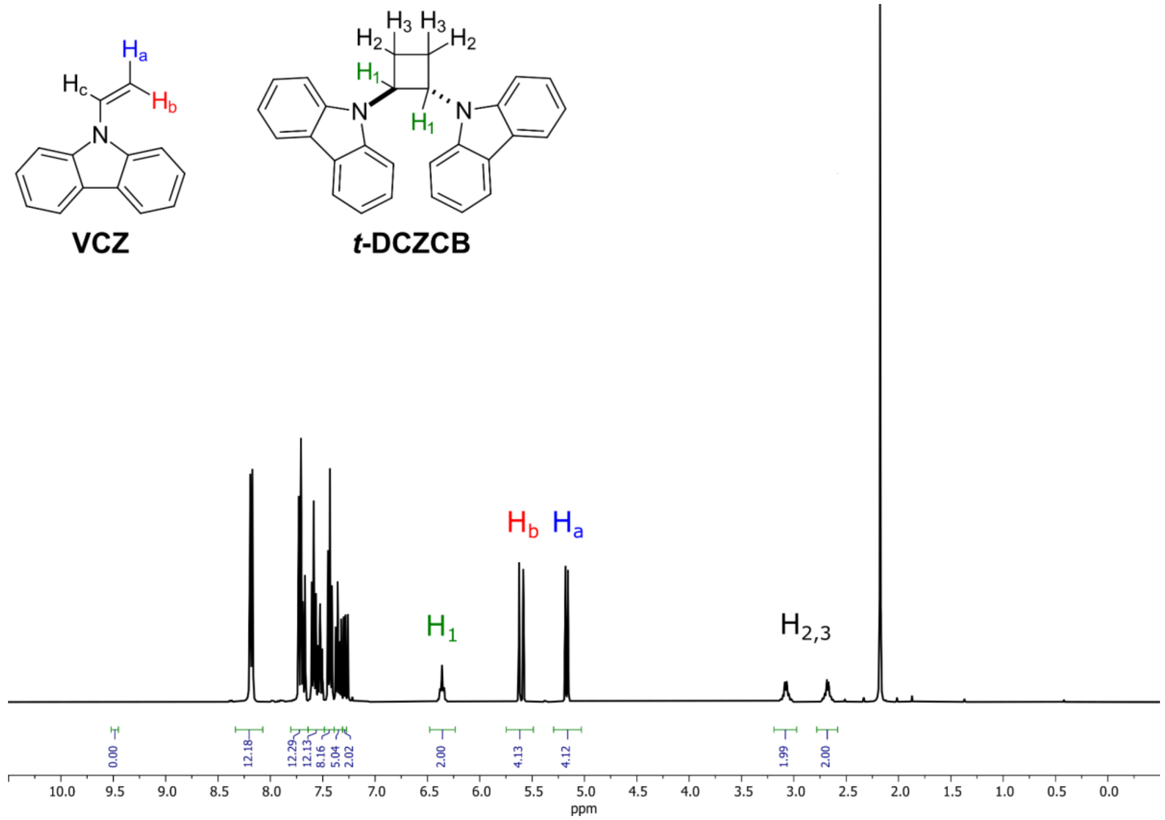

Figure S22. 400 MHz ^{1}H NMR spectrum of ***t*-DCZCB** product mixture in $CDCl_3$. Measurement taken of 6 mL aliquot during the last hour of the 18 hour continuous flow experiment.

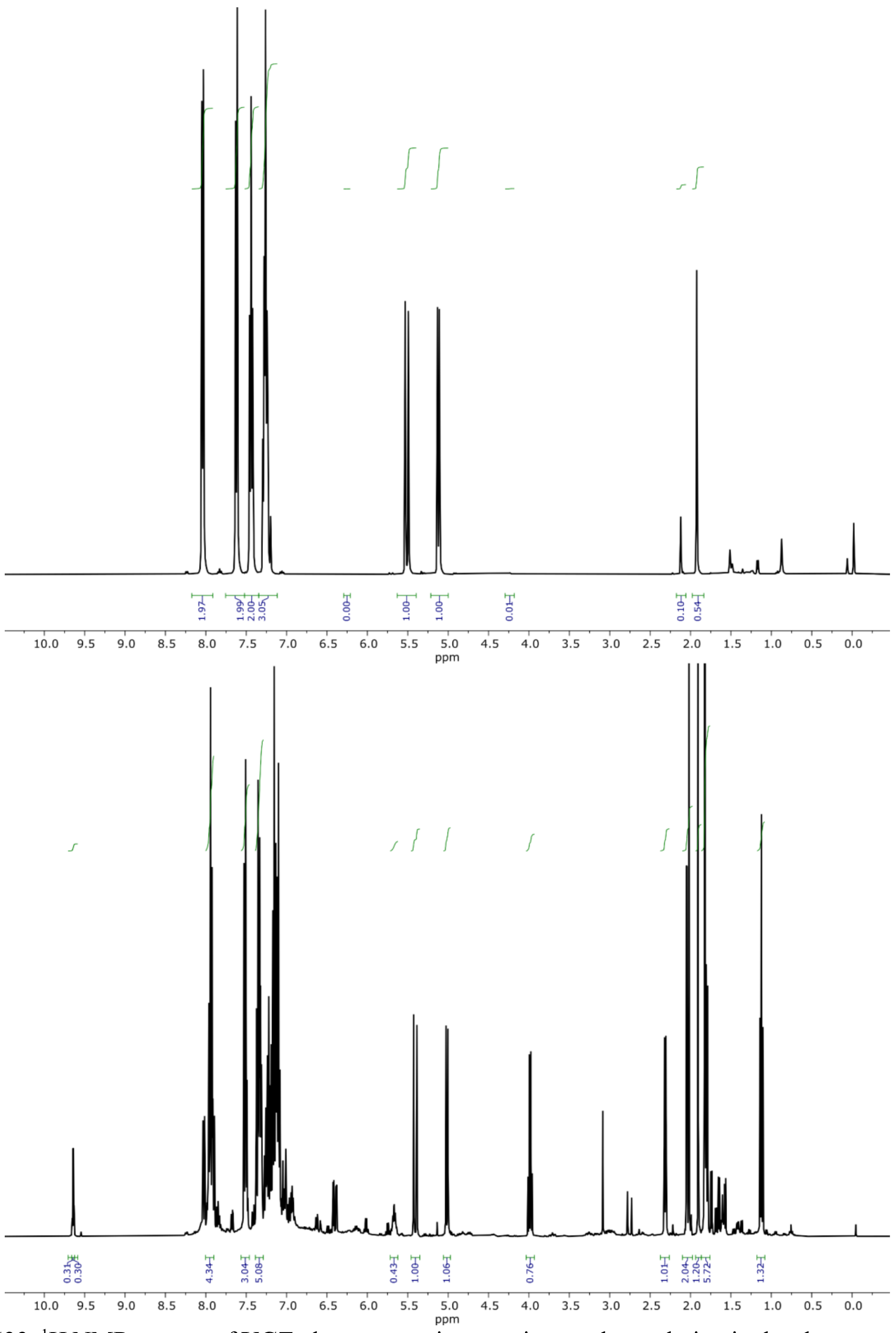

Figure S23. ^{1}H NMR spectra of **VCZ** photoconversion reaction product solution in the absence of catalyst with: (top) 525 nm and (bottom) 440 nm light irradiation. The residence times of the reactions for the top and bottom spectra were 30.12 min and 15.15 min, respectively.

To estimate the total reaction conversion and the grams per hour from this continuous manufacturing study, a Jupyter notebook is supplied as a supporting document which has advanced data analysis (filename: photochem_conversion.ipynb). The code from this notebook was written so that it can be executed from a Google Colaboratory environment. To run these code cells, the csv file with the conversion data (filename: flowchem_nmr_data.csv) should first be loaded into Google Drive and then the .ipynb file can be opened in Colab and executed to run the script. This file can also be used for other similar workflows for data analysis

In short, the Python script:

1. Opens the data, converts data to a pandas dataframe, and plots it for visualization
2. Drops data points below 45 minutes (which is not yet in steady-state), and the datapoint below 0% conversion (just below 600 minutes).
3. Calculates the area under the curve using Simpson's rule, which uses each point in the dataset to estimate the area under the conversion curve. Area: 62750.90 (minutes·conv)
4. Calculates the theoretical maximum conversion for this time. Area: 102233.13 (minutes·conv)
5. Fits a 2nd order polynomial (rather than use Simpson's rule) and estimates the area under the polynomial curve. Area 62754.20 (minutes·conv)
6. Fits a 3rd order polynomial (rather than use Simpson's rule) and estimates the area under the polynomial curve. Area 62754.34 (minutes·conv)
7. Determines the total time elapsed. Time: 17.04 hrs
8. Calculates the theoretical maximum grams over the total time. Grams: 6.816 grams over 17.04 hrs
9. Calculates the actual grams over the total time. Actual: 4.18 grams over 17.04 hrs
10. Calculates the percentage of the theoretical max: Actual: 61.38%

9. Continuous Flow Catalyst Leaching Experiment

To test the leaching of **PPEST** from PFA tubing under continuous flow, a typical experiment was designed in which photocatalyst leaching into the reaction product mixture would be confirmed by fluorescence spectroscopy of the crude product solution. A reaction of **VCZ** with **PPEST** was performed at 0.3 Watts radiant power, 525 nm (green) light, and 15.15 min τ_{res}. The concentration of **VCZ** was 50 mg·mL^{-1} (0.26 M) in acetone and 16 mL of the product solution was collected. The emission spectrum of the crude solution was compared to **PPEST**, **VCZ**, and ***t*-DCZCB** (Figure S24) showing no dye leaching during this experiment.

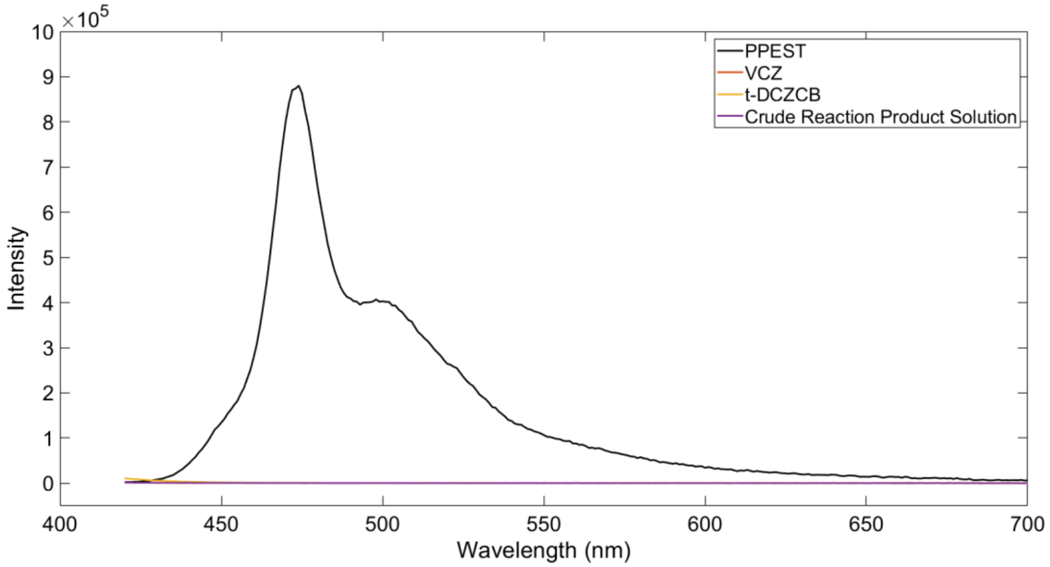

Figure S24. Emission spectra of **PPEST**, **VCZ**, ***t*-DCZCB**, and product mixture without **PPEST** in acetone for the continuous flow leaching experiment. Excitation wavelength: 350 nm. No photocatalyst was detected in the product solution.

10. NMR Spectra

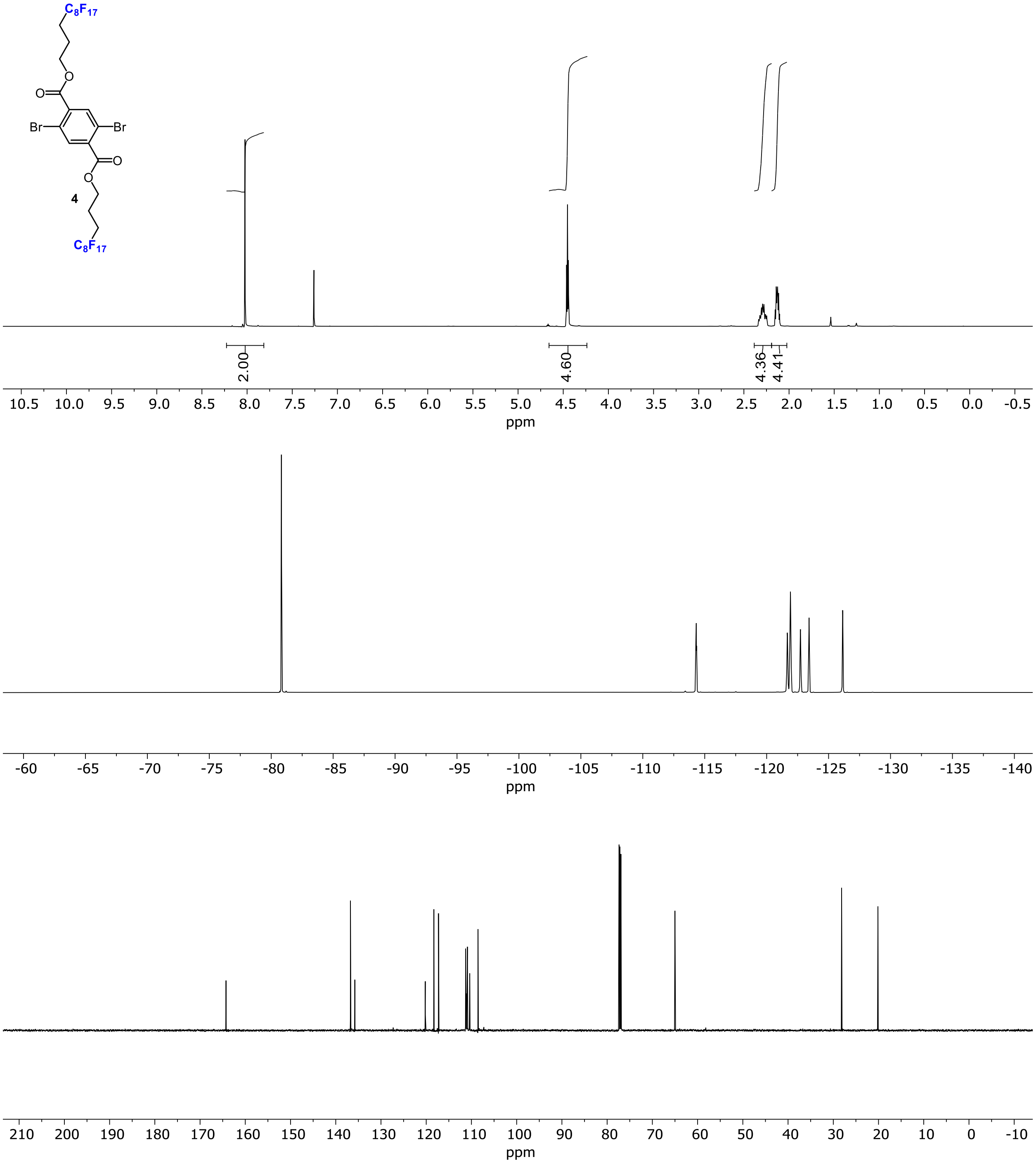

Figure S25. ^{1}H, ^{19}F and ^{13}C NMR spectra of compound **4**.

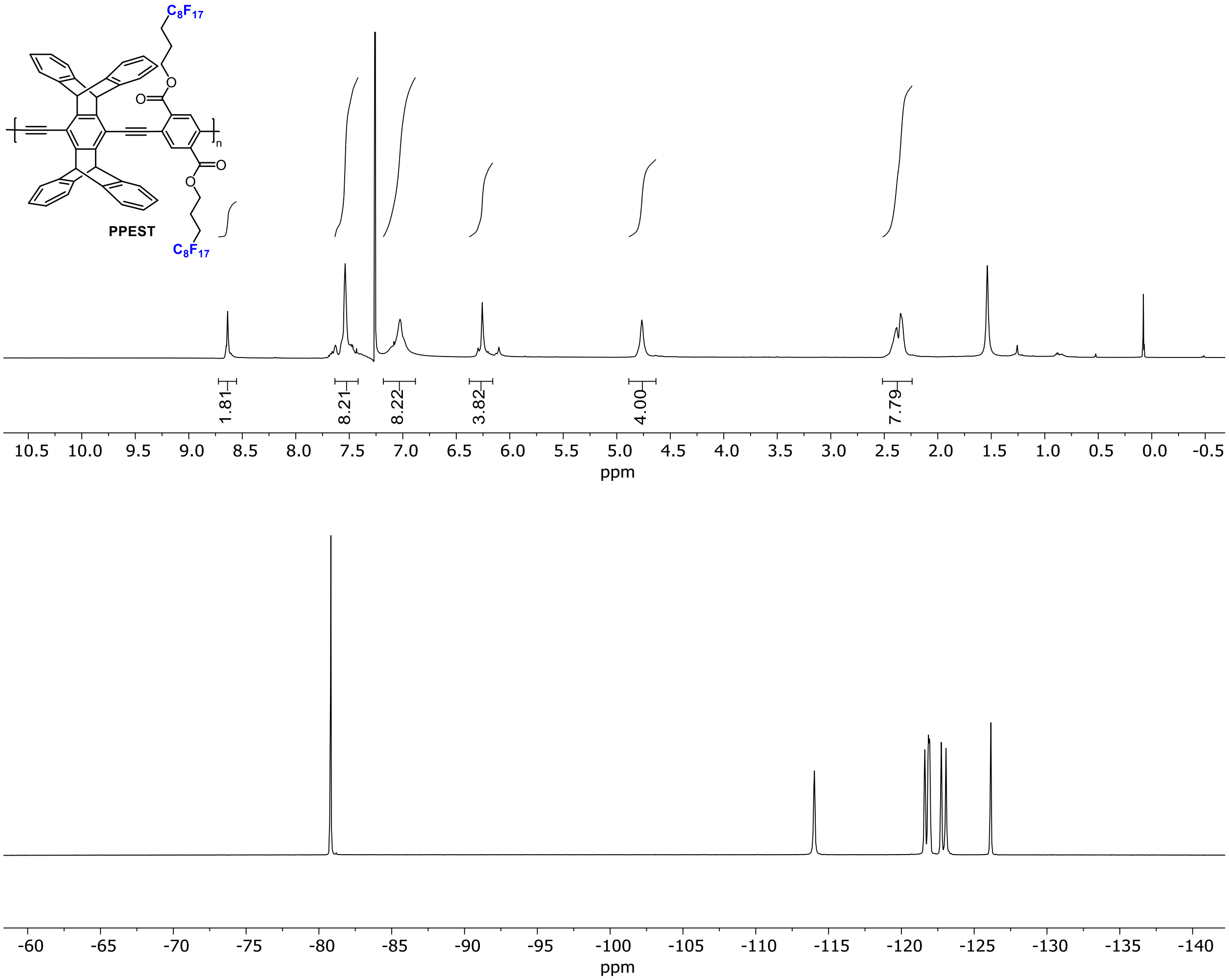

Figure S26. ^{1}H and ^{19}F NMR spectra of compound **PPEST**.

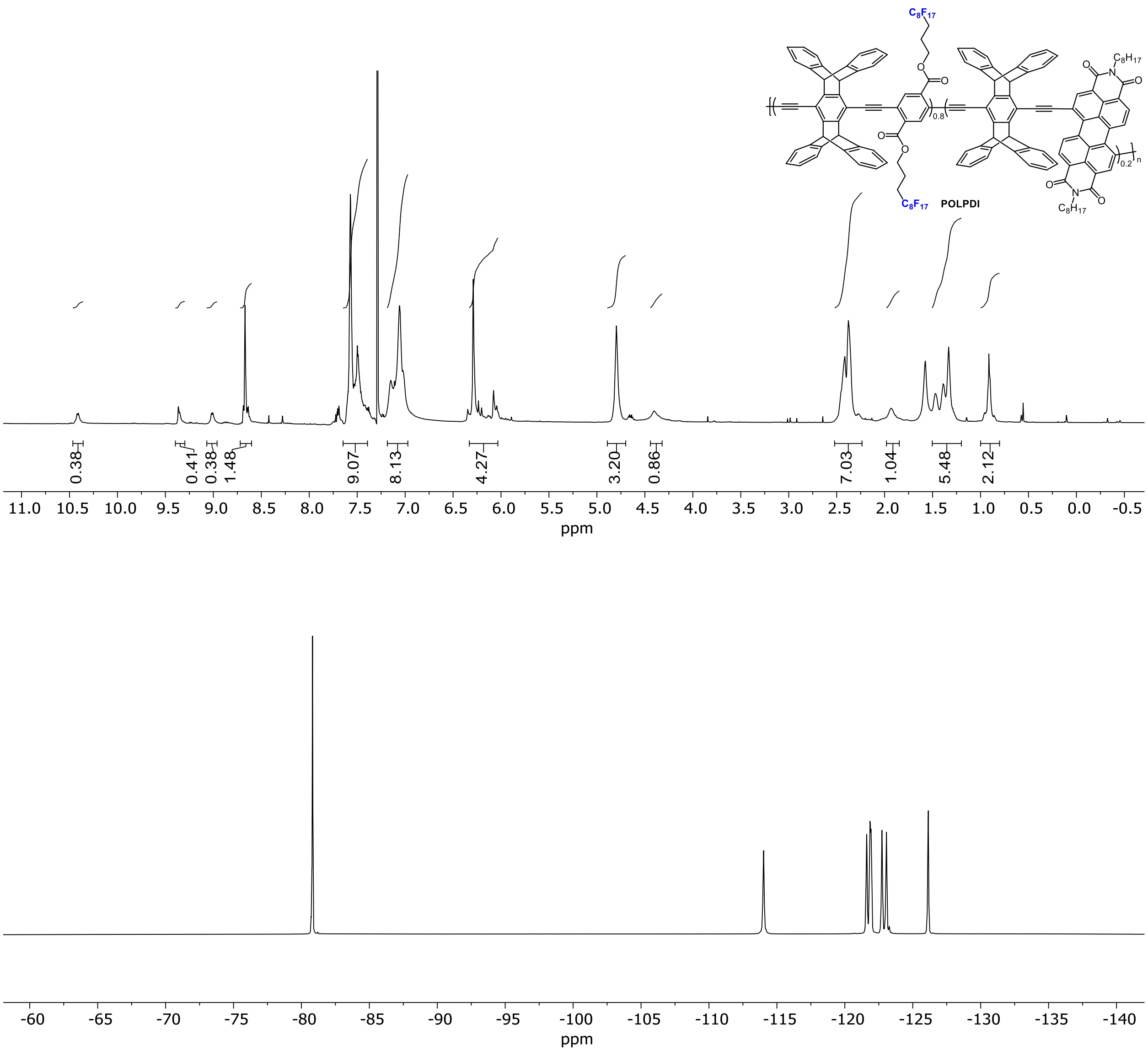

Figure S27. ^{1}H and ^{19}F NMR spectra of compound **POLPDI**.

11. Alternative Reactions Studied

To study the utility of the solid-supported photocatalyst method used in this study, alternative photochemical reactions were tested using **PPEST** or **F-PDI** (the most stable photocatalysts in this work) under green and blue light irradiation. A similar procedure for the iodoperfluoroalkylation of alkenes, reported by Prato, Kappe and coworkers[4], was tested as well as a reported procedure for amine α-alkylation of tetrahydroisoquinolines by Stephenson and coworkers.[5]

Perfluorobutylation of Methyl 10-Undecenoate

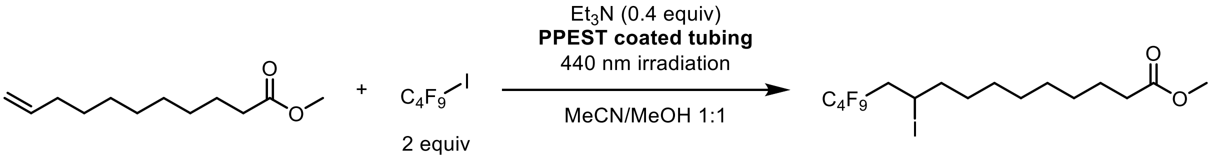

The reaction was tested using a freshly-coated PFA tube reactor with **PPEST** and the flow conditions described previously. In brief, the parameters for both 525 nm and 440 nm irradiation were: 3.0 watts radiant power (maximum power), 25 ºC, 15.15 min τ_{res}, and a collection volume of 2.5 mL. The reaction solvent was a 1:1 v/v Acetonitrile:Methanol mixture, and the reagent solution was sparged and kept under a positive pressure of argon during the reaction. The reagent solution was prepared as follows: methyl 10-undecenoate (3 mmol, 1 eq.) was mixed into 24 mL of the solvent mixture. Triethylamine (1.2 mmol, 0.4 eq.) was then added, followed by nonafluorobutyl iodide (6 mmol, 2 eq.). The solution was then sparged with argon for 5 minutes and kept under argon for the duration of the reaction. The perfluorobutylation product was quantified by ^{1}H NMR spectroscopy with 1,3-dinitrobenzene (0.0258 g, 0.15 mmol) as an internal standard.

For 525 nm light condition, no product conversion was identified by ^{1}H NMR (Figure S30) even after nearly 3 hours of continuous flow. Additionally, no apparent leaching of **PPEST** from the PFA tubing was observed by eye following the reaction, as seen in Figure S28 below. Performing the reaction under 440 nm irradiation, however, gave 0.028 mmol product (8.99 % yield relative to the internal standard), but also resulted in loss of the **PPEST** coating from a single flow reaction (~60 minutes of flow). We note that the low conversion is likely due to leaching of catalyst prior to the reaction reaching steady-state conditions, as the first 50 min (3x residence times) are diverted to waste before sample collection.

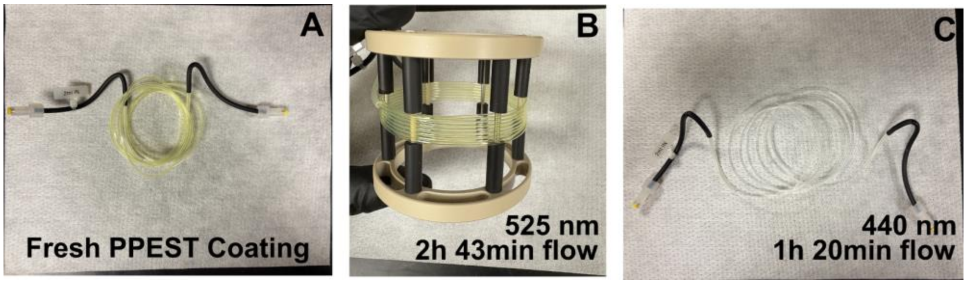

Figure S28. PFA tubing used for perfluorobutylation reactions. (A) PFA tubing with un-used **PPEST** coating, (B) The same reactor after 2 hours 43 minutes of flow with 525 nm light irradiation showing retention of the catalyst coating, and (C) the same reactor after 1 hour 20 minutes of flow with 440 nm light irradiation, showing degradation or loss of the catalyst.

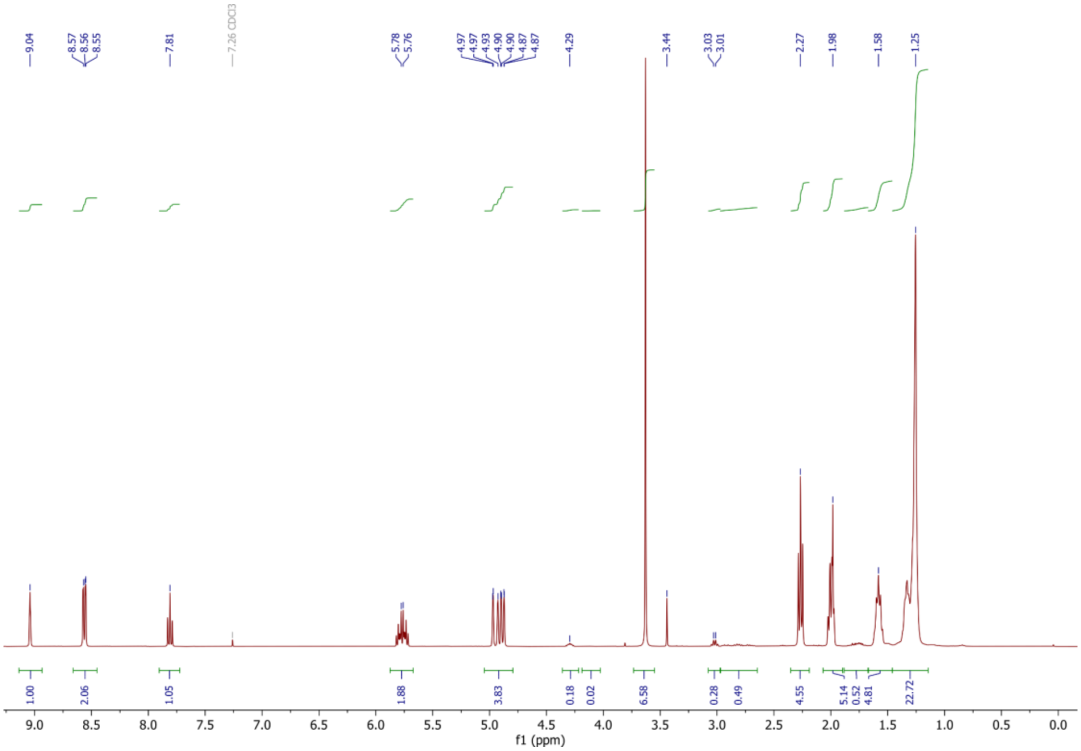

Figure S29. 400 MHz ^{1}H NMR spectrum of the perfluorobutylation crude product mixture in $CDCl_3$ after reaction with **PPEST** under 440 nm irradiation. 1,3-dinitrobenzene was added as internal standard (chemical shifts: 9 ppm, 8.56 ppm, 7.81 ppm).

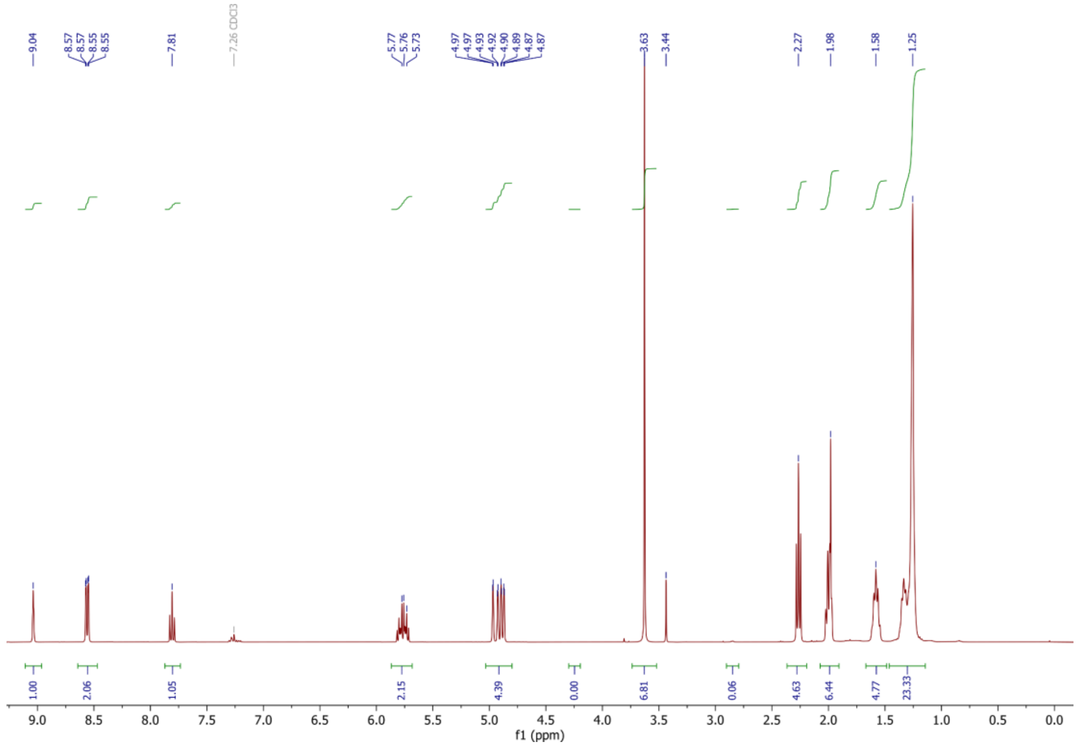

Figure S30. 400 MHz ^{1}H NMR spectrum of the perfluorobutylation crude product mixture in $CDCl_3$ after reaction with **PPEST** under 525 nm irradiation. 1,3-dinitrobenzene was added as internal standard (chemical shifts: 9 ppm, 8.56 ppm, 7.81 ppm). No product was detected.

Characterization data of methyl 12,12,13,13,14,14,15,15,15-nonafluoro-10-iodopentadecanoate:

A sample of product suitable for analysis was obtained after silica gel column chromatography of an aliquot of the crude reaction mixture using hexanes/CH_2Cl_2 (6:1 to 3:1) as eluent.
^{1}H NMR (600 MHz, $CDCl_3$) δ: 4.39 – 4.22 (m, 1H), 3.66 (s, 3H), 2.91 (ddt, *J* = 31.7, 15.7, 6.1 Hz, 1H), 2.77 (ddt, *J* = 30.9, 15.7, 7.8 Hz, 1H), 2.30 (t, *J* = 7.5 Hz, 2H), 1.90 – 1.72 (m, 2H), 1.67 – 1.60 (m, 2H), 1.57 – 1.48 (m, 1H), 1.46 – 1.21 (m, 9H). **^{19}F NMR** (565 MHz, $CDCl_3$) δ: -81.03 (q, *J* = 10.2 Hz, 3F), -110.89 – -112.44 (m, 1F), -113.62 – -115.48 (m, 1F), -124.34 – -124.93 (m, 2F), -125.12 – -126.39 (m, 2F). **^{13}C NMR** (151 MHz, $CDCl_3$, carbons directly bound to fluorine were not visible in this spectrum due to complex C-F coupling patterns) δ: 174.43, 51.60, 41.72 (t, *J* = 20.8 Hz), 40.45, 34.22, 29.66, 29.29, 29.26, 29.20, 28.56, 25.05, 20.92 ppm. **HRMS** (DART) *m/z* calculated for $C_{16}H_{23}O_2F_9$ I $[M+H]^+$, 545.05935; found, 545.05916.

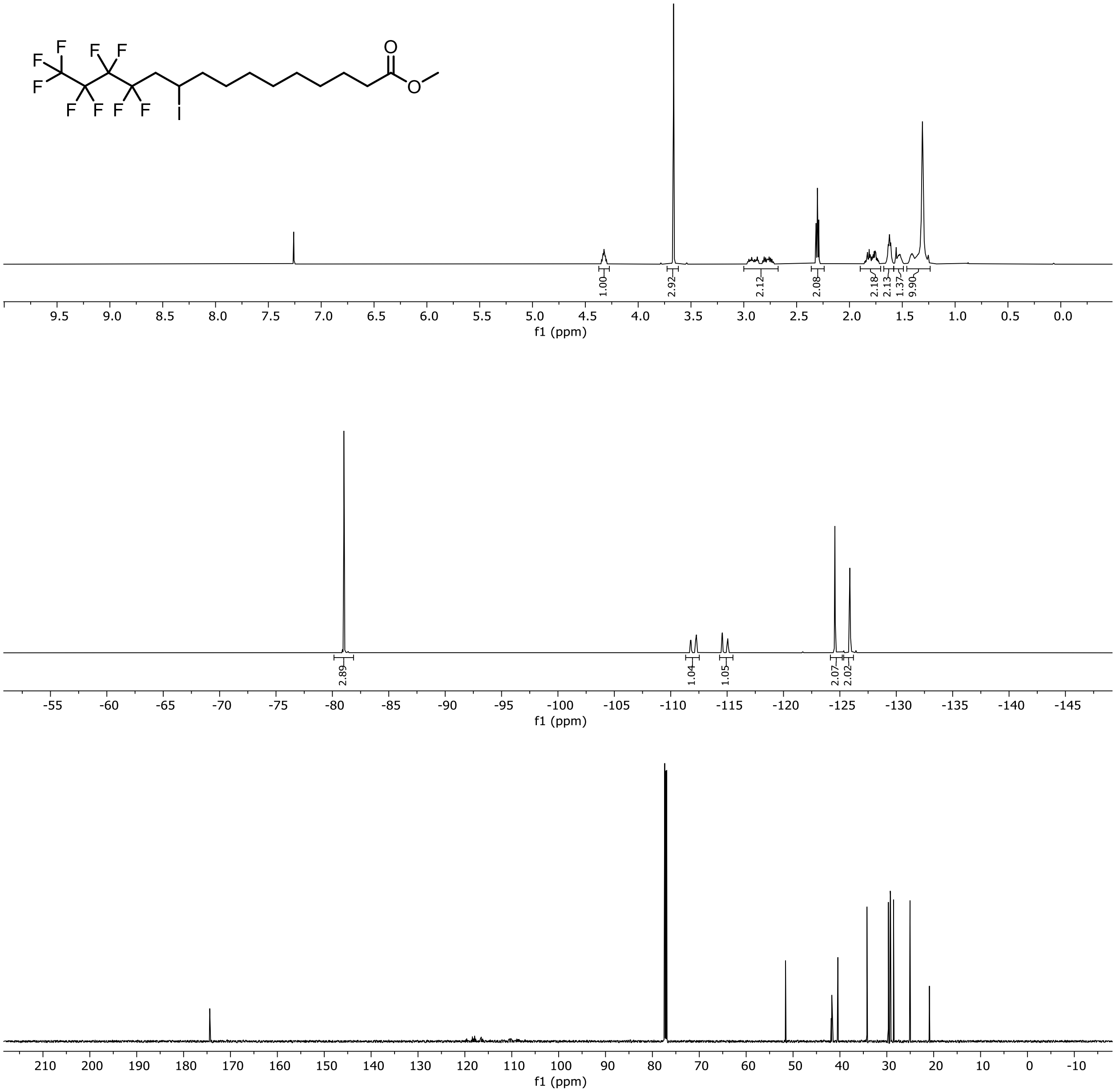

Figure S31. ^{1}H, ^{19}F and ^{13}C NMR spectra of the perfluorobutylation product.

Amine α-Alkylation of 2-Phenyl-1,2,3,4-tetrahydroisoquinoline

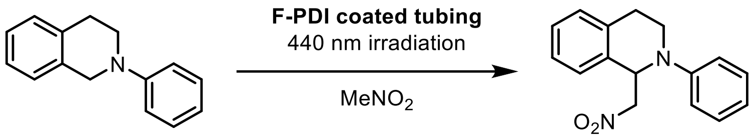

To further test potential reactions for which the fluorous polymers on PFA might be applicable, the Aza-Henry reaction of 2-Phenyl-1,2,3,4-tetrahydroisoquinoline (**THIQ**) with nitromethane ($MeNO_2$) was identified as a candidate. The reaction was tested on a new **F-PDI**-coated PFA tube reactor using the same flow conditions as the perfluorobutylation described above, with the exception of the solvent ($MeNO_2$) and photocatalyst (**F-PDI**). The **THIQ** reagent was prepared as a 10 $mg \cdot mL^{-1}$ solution in $MeNO_2$. A control experiment was also performed in the absence of photocatalyst for both LED lamps. The conversions were calculated based on ^1H NMR spectroscopy measurements of the crude reaction mixtures (Figure S32-33), and conversions for each condition are provided in Table S4 below.

Table S4. Photocatalyzed α-alkylation of **THIQ** by **F-PDI** in continuous flow for green (525 nm) and blue (440 nm) LEDs light.

525 nm, F-PDI	525 nm, No Catalyst	440 nm, F-DPI	440 nm, No Catalyst
1.96 %	0.99 %	3.38 %	3.85 %

As seen in the Table S4 above, the α-alkylation of **THIQ** is quite low for the conditions tested. Additionally, there is a significant degree of conversion without photocatalyst present. Further experiments could focus on testing other photocatalysts (**PPEST**, **POLPDI**) and further optimizing the photoreaction for lamp power, residence time, etc.

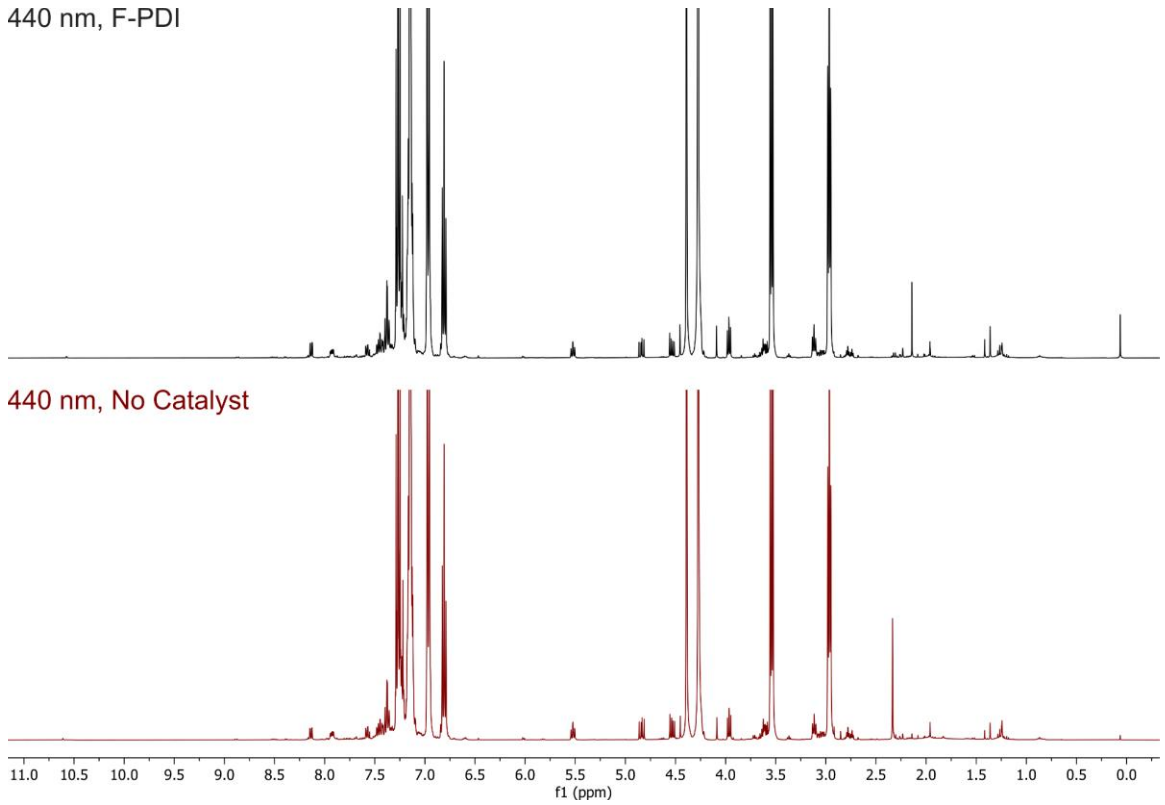

Figure S32. 400 MHz ^{1}H NMR spectrum of the nitrated **THIQ** product mixture obtained under continuous flow with 440 nm light in the presence of **F-PDI** catalyst (top) and absence of catalyst (bottom). Samples were concentrated and dissolved in $CDCl_3$ prior to NMR spectroscopy measurements.

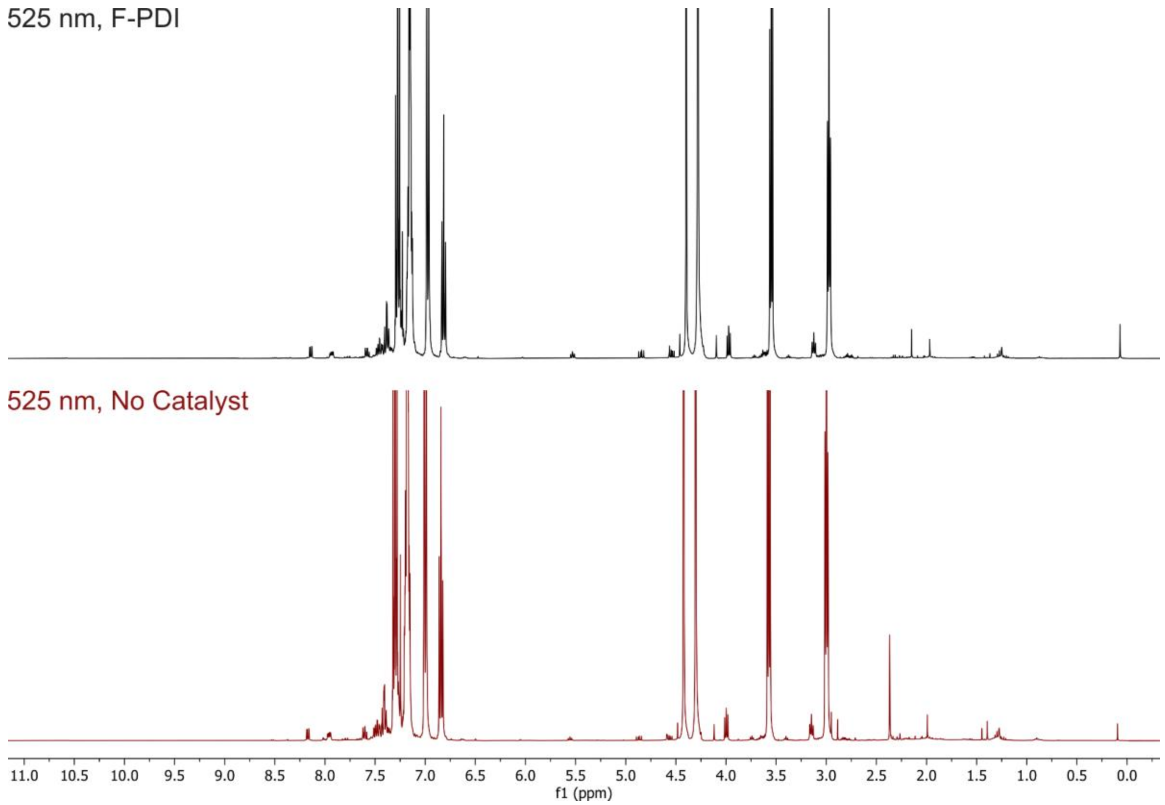

Figure S33. 400 MHz ^{1}H NMR spectrum of the nitrated **THIQ** product mixture obtained under continuous flow with 525 nm light in the presence of **F-PDI** catalyst (top) and absence of catalyst (bottom). Samples were concentrated and dissolved in $CDCl_3$ prior to NMR spectroscopy measurements.

12. References

1. Yoshinaga, K.; Swager, T. M. Fluorofluorescent Perylene Bisimides. *Synlett* **2018**, *29*, 2509-2514.
2. Yang, J.S.; Swager, T. M. Fluorescent Porous Polymer Films as TNT Chemosensors: Electronic and Structural Effects. *J. Am. Chem. Soc.* **1998**, *120*, 11864-11873.
3. Dunlap, J. H.; Ethier, J. G.; Putnam-Neeb, A. A.; Iyer, S.; Luo, S.-X. L.; Feng, H.; Garrido Torres, J. A.; Doyle, A. G.; Swager, T. M.; Vaia, R. A.; Mirau, P.; Crouse, C. A.; Baldwin, L. A. Continuous flow synthesis of pyridinium salts accelerated by multi-objective Bayesian optimization with active learning. *Chem. Sci*. **2023**, *14*, 8061-8069.
4. Rosso, C.; Williams, J. D.; Filippini, G.; Prato, M.; Kappe, C. O. Visible-Light-Mediated Iodoperfluoroalkylation of Alkenes in Flow and Its Application to the Synthesis of a Key Fulvestrant Intermediate. *Org. Lett.* **2019**, *21*, 5341-5345.
5. Condie, A. G.; González-Gómez, J. C.; Stephenson, C. R. J. Visible-Light Photoredox Catalysis: Aza-Henry Reactions via C-H Functionalization. *J. Am. Chem. Soc.* **2010**, *132*, 1464-1465.
